# Susceptibility factor StEXA1 interacts with StnCBP to facilitate potato virus Y accumulation through the stress granule-dependent RNA regulatory pathway in potato

**DOI:** 10.1093/hr/uhac159

**Published:** 2022-07-22

**Authors:** Ruhao Chen, Zhen Tu, Changzheng He, Xianzhou Nie, Kun Li, Sitian Fei, Botao Song, Bihua Nie, Conghua Xie

**Affiliations:** Key Laboratory of Potato Biology and Biotechnology (HZAU), Ministry of Agriculture and Rural Affairs, Key Laboratory of Horticultural Plant Biology (HZAU), Ministry of Education, Huazhong Agricultural University, Wuhan, 430070, China; ERC for Germplasm Innovation and New Variety Breeding of Horticultural Crops, Key Laboratory for Vegetable Biology of Hunan Province, Hunan Agricultural University, Changsha, 410128, China; Key Laboratory of Potato Biology and Biotechnology (HZAU), Ministry of Agriculture and Rural Affairs, Key Laboratory of Horticultural Plant Biology (HZAU), Ministry of Education, Huazhong Agricultural University, Wuhan, 430070, China; ERC for Germplasm Innovation and New Variety Breeding of Horticultural Crops, Key Laboratory for Vegetable Biology of Hunan Province, Hunan Agricultural University, Changsha, 410128, China; Fredericton Research and Development Centre, Agriculture and Agri-Food Canada, Fredericton, New Brunswick, E3B 4Z7, Canada; Key Laboratory of Potato Biology and Biotechnology (HZAU), Ministry of Agriculture and Rural Affairs, Key Laboratory of Horticultural Plant Biology (HZAU), Ministry of Education, Huazhong Agricultural University, Wuhan, 430070, China; Key Laboratory of Potato Biology and Biotechnology (HZAU), Ministry of Agriculture and Rural Affairs, Key Laboratory of Horticultural Plant Biology (HZAU), Ministry of Education, Huazhong Agricultural University, Wuhan, 430070, China; Key Laboratory of Potato Biology and Biotechnology (HZAU), Ministry of Agriculture and Rural Affairs, Key Laboratory of Horticultural Plant Biology (HZAU), Ministry of Education, Huazhong Agricultural University, Wuhan, 430070, China; Key Laboratory of Potato Biology and Biotechnology (HZAU), Ministry of Agriculture and Rural Affairs, Key Laboratory of Horticultural Plant Biology (HZAU), Ministry of Education, Huazhong Agricultural University, Wuhan, 430070, China; Key Laboratory of Potato Biology and Biotechnology (HZAU), Ministry of Agriculture and Rural Affairs, Key Laboratory of Horticultural Plant Biology (HZAU), Ministry of Education, Huazhong Agricultural University, Wuhan, 430070, China

## Abstract

Plant viruses recruit multiple host factors for translation, replication, and movement in the infection process. The loss-of-function mutation of the susceptibility genes will lead to the loss of susceptibility to viruses, which is referred to as ‘recessive resistance’. *Essential for potexvirus Accumulation 1* (*EXA1)* has been identified as a susceptibility gene required for potexvirus, lolavirus, and bacterial and oomycete pathogens. In this study, *EXA1* knockdown in potato (*StEXA1*) was found to confer novel resistance to potato virus Y (PVY, potyvirus) in a strain-specific manner. It significantly compromised PVY^O^ accumulation but not PVY^N:O^ and PVY^NTN^. Further analysis revealed that StEXA1 is associated with the HC-Pro of PVY through a member of eIF4Es (StnCBP). HC-Pro^O^ and HC-Pro^N^, two HC-Pro proteins from PVY^O^ and PVY^N^, exhibited strong and weak interactions with StnCBP, respectively, due to their different spatial conformation. Moreover, the accumulation of PVY^O^ was mainly dependent on the stress granules (SGs) induced by StEXA1 and StnCBP, whereas PVY^N:O^ and PVY^NTN^ could induce SGs by HC-Pro^N^ independently through an unknown mechanism. These results could explain why *StEXA1* or *StnCBP* knockdown conferred resistance to PVY^O^ but not to PVY^N:O^ and PVY^NTN^. In summary, our results for the first time demonstrate that *EXA1* can act as a susceptibility gene for PVY infection. Finally, a hypothetical model was proposed for understanding the mechanism by which StEXA1 interacts with StnCBP to facilitate PVY accumulation in potato through the SG-dependent RNA regulatory pathway.

## Introduction

Potato (*Solanum tuberosum* L.) has become the third most important food crop worldwide next to rice and wheat in terms of human consumption [1, 2]. Over recent decades, potato cultivation has been rapidly increasing in developing countries in tropical and subtropical regions, and shows a tendency of further expanding to warmer regions of the tropics in the future [3]. However, the degradation of seed potatoes due to the accumulation of viruses caused by high temperature will massively reduce production, which may account for ≥50% of the total potential yield [4, 5].

Among the over 50 viruses reported to infect potato under field conditions [3, 6], potato virus Y (PVY), a type member of the genus *Potyvirus,* is currently a major damaging and economically important virus infecting potatoes worldwide, which can cause a yield loss of up to 80% [7]. PVY is transmitted by aphids non-persistently and infects several important crops in the *Solanaceae* family, including potato, tobacco, pepper, and tomato [7, 8]. This virus exhibits high strain/variant diversity at the biological, genetic, and molecular levels, including conventional (nonrecombinant) strains such as PVY^O^ and PVY^N^, and newly emerging recombinant strains such as PVY^NTN^ and PVY^N:O^, which were mostly developed from PVY^O^ and PVY^N^ parental lineages [9, 10].

Even in countries with highly developed potato seed certification programs, PVY management is still a major challenge due to the gross underestimation of PVY incidence caused by mildly symptomatic recombinant PVY strains, asymptomatic Typhoid Mary cultivars, and late-season asymptomatic foliar infections by aphids [9, 11]. Therefore, development of PVY-resistant cultivars is the most effective and environment-friendly approach to control PVY infection in potato.

Both dominant and recessive resistances are involved in plants to various viruses. In potato, the former includes the extreme resistance (ER) and hypersensitive resistance (HR) conferred by the *R* and *N* genes, respectively [12, 13]. So far, at least ten dominant resistance genes against PVY have been mapped on potato chromosomes IV, IX, XI, and XII, some of which were introduced into potato cultivars for PVY management (e.g. *Ry_sto_*) [3, 14]. However, only a few of them were cloned, such as *Ry_sto_*, which was characterized as a TIR-NLR-encoding gene conferring an effective ER-type response recently [15]. Recessive resistance has also been a focus in research on plant-virus interaction over the past two decades, as approximately half of the alleles responsible for virus resistance in crops are recessive [16–18]. A typical plant virus encodes about 4 to 10 proteins with a limited genome size. The genes involved in recessive resistance, which are also called susceptibility genes or host factors, are often hijacked to help complete the infection cycle of corresponding viruses, and mutation in these genes will result in the loss of susceptibility to the viruses as a heritable recessive trait in plants [19, 20].

So far, most recessive resistance genes have been identified as *eukaryotic translation initiation factor (eIF) 4E* and its isoforms (hereafter referred to as *eIF4Es*), and several mutations in *eIF4Es* conferred resistance to potyviruses in a range of hosts including pepper, lettuce, Arabidopsis, pea, and tomato [21–24]. The binding of VPg and HC-Pro to eIF4Es in several potyviruses *in vitro* and *in vivo* was identified as the key molecular mechanism for the eIF4Es-mediated recessive resistance to potyviruses [25–27]. However, the practical application of eIF4Es as recessive resistance genes is limited due to functional redundancy among eIF4Es or the embryo-lethal effect of their knockout [28, 29]. Over the past two decades, researchers have sought to identify several other non-eIF4Es types of promising recessive resistance genes against potyviruses, which have been reviewed in other studies [19, 20]. Hsp70, RH8, RH9, PABPs, SYP71, PVIP1, SYTA, PCaP1, SEC24A, DBP1, and IRE1/bZIP60 have been found to perform different functions in the process of turnip mosaic virus (TuMV) infection in *Arabidopsis thaliana* [30–40]. cPGK2, DBP1, and RH8 were required for efficient plum pox virus (PPV) multiplication in *A. thaliana* [31, 39, 41]. RAV2 and rgs-CaM are associated with tobacco etch virus (TEV) HC-Pro to suppress gene silencing in tobacco [42, 43].

However, little is known about the possible role of recessive resistance genes against PVY in potato so far. Here, we report a promising recessive resistant gene, *StEXA1,* and its underlying mechanism involved in PVY accumulation in potato.

*StEXA1* is a putative orthologue of the *A. thaliana* susceptibility gene called *Essential for poteXvirus Accumulation 1* (*AtEXA1*). AtEXA1 was first identified as a glycine-tyrosine-phenylalanine (GYF) domain-containing protein required for the infection of three potexviruses (*plantago asiatica* mosaic virus, PlAMV; alternanthera mosaic virus, AltMV; and potato virus X, PVX) [44]. Further research suggested that the function of *EXA1* orthologue(s) in tobacco and tomato is highly conserved in the infection of several potexviruses and a lolavirus [45]. Moreover, two other studies have reported that *EXA1* is also involved in plant immune responses to bacterial and oomycete pathogens in *A. thaliana* [46,47]. Although *EXA1* encodes >1700 amino acids, only two small conserved functional domains have been identified, including an eIF4E-binding motif and a GYF domain. The former consists of Tyr-X-X-X-X-Leu-phi (YXXXXLΦ) binding to eIF4Es, while the latter is an adapter binding to proline-rich sequences (PRSs) [48, 49]. The *EXA1* gene plays an important role in the infections of various types of pathogens. However, it remains elusive how it functions as a recessive resistance gene. In this study, we show that *StEXA1* knockdown compromised PVY accumulation in a strain-specific manner, whereas it did not affect PVX and potato virus M (PVM) accumulation in potato. Our results also demonstrated that StEXA1 can interact with a potato novel cap-binding protein (StnCBP), which is recognized by the HC-Pro proteins of PVY^O^ and PVY^N:O/NTN^ with different interaction strengths. Moreover, we found that StEXA1 and StnCBP may participate in the assembly of stress granules (SGs) as potential components, and PVY may need to hijack the SG-dependent RNA regulatory process to facilitate its infection in potato.

## Results

### *StEXA1* knockdown significantly compromised PVY^O^ accumulation

We used the amino acid sequence of AtEXA1 (AT5G42950) protein as a query to perform a BLASTp search against the potato reference genome *S. tuberosum* group Phureja DM1–3 v6.1 (Spud DB). A unique GYF domain-containing protein Soltu.DM.04G035210 (hereafter referred to as StEXA1) was identified in potato, which showed a 43.4% amino acid sequence identity with AtEXA1. A phylogenetic tree was constructed with the amino acid sequences of 24 GYF domain-containing proteins from *A. thaliana*, *S. tuberosum*, *Nicotiana benthamiana,* and *Solanum lycopersicum*. Obviously, the four EXA1 orthologues were clustered together, representing a specific class of GYF domain-containing proteins (see online  supplementary data Fig. S1), and StEXA1 showed higher amino acid sequence identities with tobacco NbEXA1 and tomato SlEXA1 (81.1% and 91.4%, respectively) (see online supplementary data Fig. S2). Similar to other EXA1 proteins reported, StEXA1 encodes 1715 amino acids and also comprises the two conserved domains: an eIF4E-binding motif from the 298th to 304th amino acid residue and a GYF domain from the 533rd to 590th amino acid residue (see online supplementary data Fig. S2).

Then, the full length *StEXA1* was cloned from Eshu3 and submitted to GenBank (accession number: ON798805), it shared approximately 99.9% nucleotide identity with that from Phureja DM1–3 (see online supplementary data Fig. S3a). To silence *StEXA1* by RNA interference (RNAi), a 327-bp region in the 5′-terminus of *StEXA1* (from Eshu 3) was selected as the RNAi target, which returned no other homologous genes except for *StEXA1* in a BLASTn search against Spud DB. Three transgenic lines with high interference efficiency (designated as RiStEXA1–1, RiStEXA1–2, and RiStEXA1–3) (see online supplementary data Fig. S4a) were then obtained by *Agrobacterium*-mediated transformation in the potato cultivar Eshu 3 and used for further research. The potential target genes of the 327-bp region were evaluated using a sequence complementarity-based approach as described previously [50]. Among the resultant 381 genes predicted as potential targets (see online supplementary data Table S1), only *StEXA1* (Soltu.DM.04G035210) was found in the 106 significantly down-regulated genes in RiStEXA1 lines according to the RNA sequencing data (supplementary data Table S2 and see online Fig. S5). These results indicated no occurrence of the off-target event in the transgenic lines.

**Figure 1:**
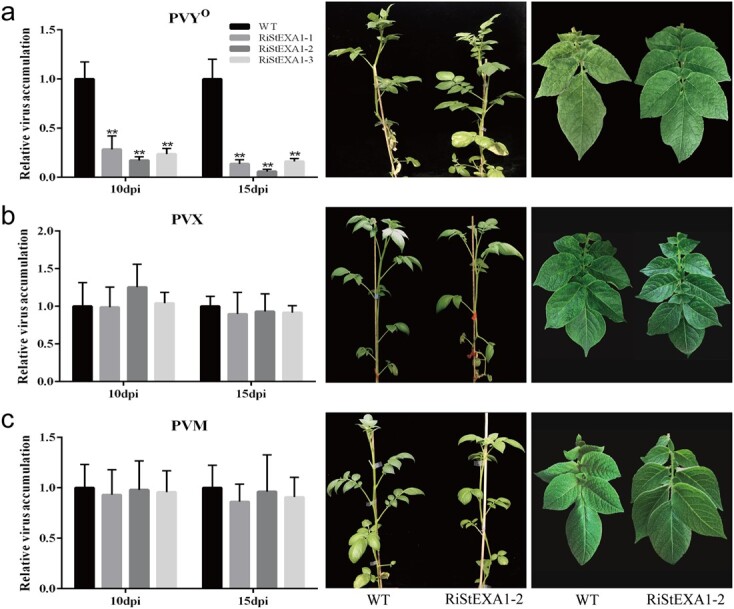
The viruses accumulate and symptoms develop in the RiStEXA1 and control plants (WT) inoculated with PVY^O^ (**a**), PVX (**b**), and PVM (**c**). Relative virus accumulation was determined using qRT–PCR with total RNAs extracted from the non-inoculated upper leaves at 10 and 15 dpi. Symptoms on plants and systemic leaves were observed and photos were taken at ~25 dpi. Data are presented as means ± SD (*n* = 3) relative to WT plants, and *EF1α* was used as the normalizer. Three independent experiments were performed with similar results. Asterisks indicate statistically significant differences according to Student’s *t* test (^**^P < 0.01).

**Figure 2:**
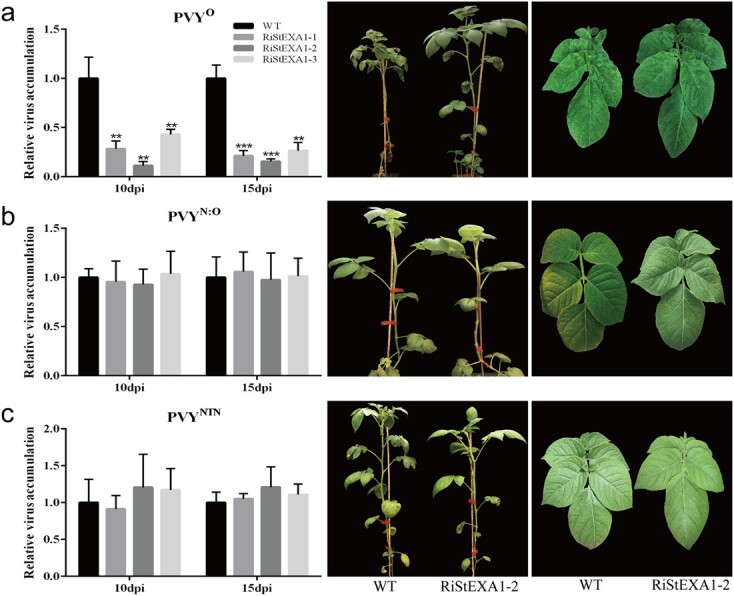
The viruses accumulate and symptoms develop in the RiStEXA1 and control plants (WT) inoculated with PVY^O^ (**a**), PVY^N:O^ (**b**), and PVY^NTN^ (**c**). Relative virus accumulation was determined using qRT–PCR with total RNAs extracted from the non-inoculated upper leaves at 10 and 15 dpi. Symptoms on plants and systemic leaves were observed and photos were taken at ~25 dpi. Data are presented as means ± SD (*n* = 3) relative to WT plants, and *EF1α* was used as the normalizer. Three independent experiments were performed with similar results. Asterisks indicate statistically significant differences according to Student’s *t* test (^***^P < 0.001, ^**^P < 0.01).

The selected transgenic lines were then inoculated with several potato viruses including PVY (isolate PVY^O^-FL, hereafter referred to as PVY^O^), PVX, and PVM to test whether *StEXA1* knockdown would cause loss of susceptibility to potato viruses. Interestingly, only PVY^O^ accumulation was significantly suppressed in the three transgenic lines, as indicated by the reduction in the severity of the mosaic symptoms and viral titre determined using quantitative RT-PCR and ELISA assays in the upper systemic leaves at 10 and 15 days post-inoculation (dpi), while PVX and PVM still readily infected the transgenic plants as in the control plants (WT) (Fig. 1 and online supplementary data Fig. S6a). These results indicate that *StEXA1* is essential for PVY infection. Therefore, we further tested the virus resistance of the transgenic lines after inoculation with other strains of PVY. Surprisingly, unlike PVY^O^, the two recombinant strains (PVY^N:O^ and PVY^NTN^) successfully infected the transgenic plants as in the WT without obvious symptoms (Fig. 2 and online supplementary data Fig. S6b). Thus, the *StEXA1* gene seems to be strain-specifically involved in PVY infection in potato.

### HC-Pro protein of PVY^O^ played a vital role in the *StEXA1*-mediated recessive resistance to PVY^O^

**Figure 3:**
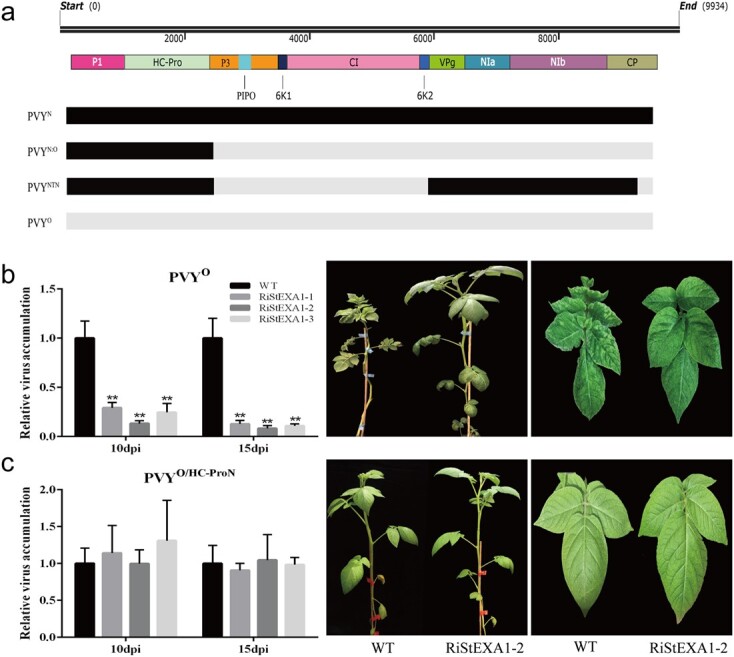
HC-Pro involves the *StEXA1-*mediated recessive resistance against PVY^O^. **a** Schematic illustration of the genomic structure of PVY^O^, PVY^N^, PVY^N:O^, and PVY^NTN^ based on their sequence sources. Black and gray bars represent the genomic sequences derived from PVY^N^ and PVY^O^, respectively. The genome size, corresponding protein names, and positions are indicated in the top row of the structural diagram. **b** and **c** The viruses accumulate and symptoms develop in the RiStEXA1 and control plants (WT) inoculated with PVY^O^ (**b**) and PVY^O/HC-ProN^(**c**). Relative virus accumulation was determined using qRT–PCR with total RNAs extracted from the non-inoculated upper leaves at 10 and 15 dpi. Symptoms on plants and systemic leaves were observed and photos were taken at ~25 dpi. Data are presented as means ± SD (*n* = 3) relative to WT plants, and *EF1α* was used as the normalizer. Three independent experiments were performed with similar results. Asterisks indicate statistically significant differences according to Student’s *t* test (^**^P < 0.01).

PVY^N:O^ and PVY^NTN^ are derivatives of PVY^O^ and PVY^N^ generated via genome recombination and possessed one and three recombinant joints (RJs), respectively. Specifically, PVY^N:O^ had an RJ located at ca. ~nt 2400 (N to O), whereas PVY^NTN^ had three RJs at ~nt 2400 (N to O), ~nt 5820 (O to N), and ~nt 9180 (N to O), respectively [51, 52]. The difference in genome sequences between PVY^O^ and the two recombinant PVY strains was located at the 5′ segment from nt 1 to ~nt 2400 (including P1 and HC-Pro protein) (Fig. 3a), which probably contributed to the resistance to PVY^O^ while having no effect on PVY^N:O^ and PVY^NTN^ infection in the RiStEXA1 lines. Subsequently, an infectious cDNA clone named PVY^O/HC-ProN^ was constructed based on the sequence of PVY^O^-FL, except that its HC-Pro protein (hereafter referred to as HC-Pro^O^) was substituted by the HC-Pro protein from PVY^N^ (hereafter referred to as HC-Pro^N^) (see online supplementary data Fig. S7). Then the inoculation assays confirmed that the susceptibility to PVY^O^ was restored in the RiStEXA1 lines due to the substitution of HC-Pro protein (Fig. 3b and c, and online supplementary data Fig. S6c). Additionally, the mosaic symptoms caused by PVY^O^ infection disappeared due to the substitution (Fig. 3c), which is consistent with the previous finding that HC-Pro protein may be involved in the symptom formation of PVY infection [53, 54]. Therefore, it can be speculated that HC-Pro^O^ is involved in the *StEXA1*-mediated recessive resistance to PVY^O^.

### StEXA1 was associated with HC-Pro protein of PVY through an eIF4Es-type protein StnCBP

To determine whether HC-Pro^O^ directly recruits StEXA1 to help PVY^O^ infection in potato, we conducted a yeast two-hybrid (Y2H) assay between StEXA1 and HC-Pro^O^, as well as all other proteins encoded by PVY^O^ (including P1, P3, PIPO, 6 K1, CI, 6 K2, VPg, NIa, NIb, and CP). Surprisingly, StEXA1 showed no direct interaction with HC-Pro^O^, or with any other proteins of PVY^O^ (see online supplementary data Fig. S8a). Both StEXA1 and HC-Pro protein encode an eIF4E-binding motif (YXXXLΦ) (see online supplementary data Fig. S9a), and *eIF4Es* were often reported as important susceptibility genes in the infection process of potyviruses. It is, therefore, reasonable to hypothesize that eIF4Es may be important host factors linking StEXA1 and HC-Pro proteins. Moreover, in the STRING database, three eIF4E proteins in potato, StnCBP (Soltu.DM.10G026730), SteIF4E (Soltu.DM.03G000970), and SteIF(iso)4E (Soltu.DM.09G027260), were predicted to interact with StEXA1 based on the interactions of the orthologous genes in other species [55–57]. The three *eIF4E* genes were then cloned from Eshu 3 and submitted to GenBank (accession numbers ON798806 for *StnCBP*, ON798804 for *SteIF4E*, and ON798803 for *SteIF(iso)4E*). The nucleotide sequences of *StnCBP*, *SteIF4E*, and *SteIF(iso)4E* from Eshu 3 shared 100%, 100%, and 99.7% identities with those from Phureja DM1–3, respectively (see online supplementary data Fig. S3b–d).

**Figure 4:**
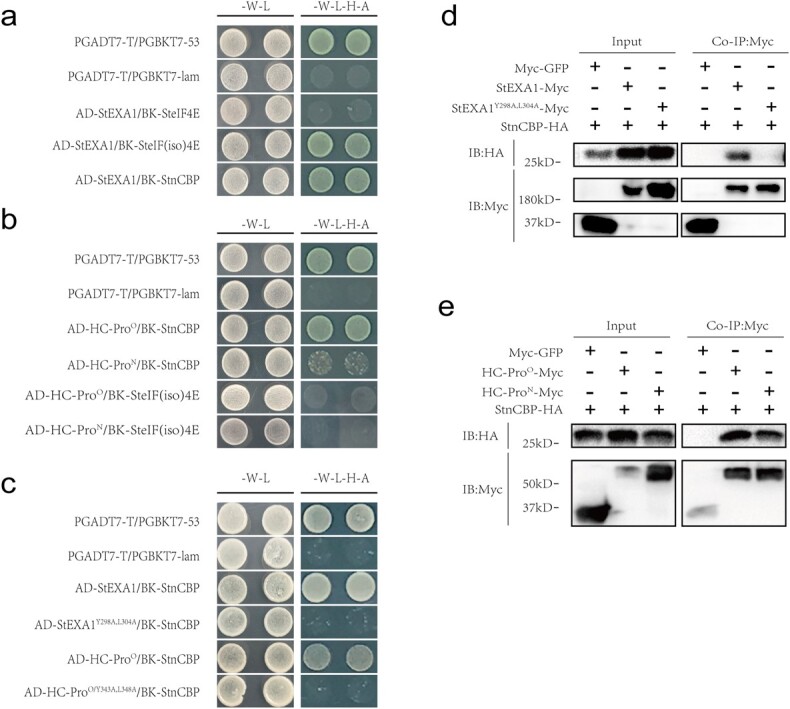
StnCBP is associated with *StEXA1-*mediated recessive resistance against PVY^O^ by recognizing HC-Pro. **a** Interactions between StEXA1 and SteIF4Es in Y2H assays. **b** Interactions between HC-Pro and SteIF4Es in Y2H assays. **c** Substitute alanine for tyrosine and leucine in eIF4E-binding motifs of StEXA1 or HC-Pro^O^ proteins abolished their interactions in Y2H assays. **d** Interactions between StEXA1/StEXA1^Y298A, L304A^ with StnCBP in Co-IP assays. **e** Interactions between HC-Pro^O^/HC-Pro^N^ and StnCBP in Co-IP assays. In **a**, **b**, and **c**, -W-L represents a medium lacking tryptophan and leucine, while -W-L-H-A represents a medium lacking tryptophan, leucine, histidine, and adenine. Paired combinations PGADT7-T/PGBKT7–53 and PGADT7-T/PGBKT7-lam represent positive and negative controls, respectively. In **d** and **e**, total proteins (input) were immunoprecipitated with anti-Myc mAb-magnetic beads (Co-IP: Myc), followed by immunoblotting using anti-Myc (IB: Myc) and anti-HA (IB: HA) antibodies.

**Figure 5:**
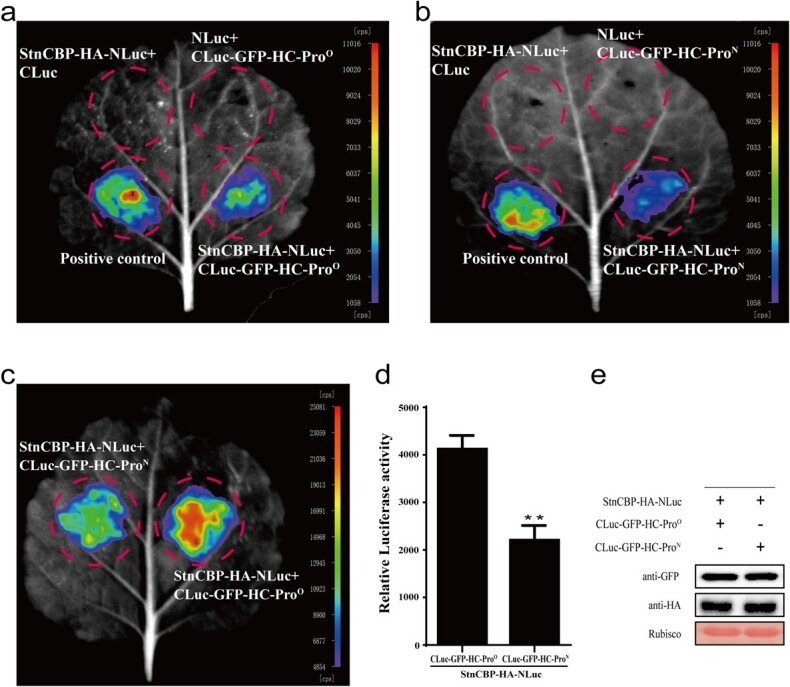
StnCBP exhibits strong and weak interactions with HC-Pro^O^ and HC-Pro^N^, respectively, in the split luciferase complementation assays in *N. benthamiana*. **a** Luminescence image of the interaction between StnCBP and HC-Pro^O^. **b** Luminescence image of the interaction between StnCBP and HC-Pro^N^. **c**, **d**, and **e** StnCBP exhibited strong and weak interactions with HC-Pro^O^ and HC-Pro^N^, respectively, under the same protein abundance. In **a**, **b**, and **c**, the luminescence images were captured using a CCD imaging system, and the pseudocolor bar shows the range of luminescence intensity. The reported interacting proteins StPHYF and StPHYB were used as the positive controls [60]. In **d**, the quantification of relative luciferase activity in leaves is equivalent to luminescence intensity/50 mm^2^ leaf area, which was calculated using IndiGO imaging software. Each bar represents the mean ± SD of three replicates (*n* = 3). Asterisks indicate statistically significant differences according to Student’s *t* test (^**^P < 0.01). In **e**, Western blotting showed the protein abundance of NLuc and CLuc fusions.

The Y2H assays were then conducted to verify the above prediction. The results confirmed that StEXA1 interacts with SteIF(iso)4E and StnCBP but not with SteIF4E (Fig. 4a). Additionally, we also tested the direct interactions of SteIF(iso)4E and StnCBP with HC-Pro^O^ and HC-Pro^N^. The results revealed that StnCBP but not SteIF(iso)4E interacts with both HC-Pro^O^ and HC-Pro^N^ (Fig. 4b), which was further demonstrated via co-immunoprecipitation (Co-IP) assays (Fig. 4d and e). Notably, StnCBP exhibited a strong interaction with HC-Pro^O^ but a weak interaction with HC-Pro^N^ in the Y2H assays (Fig. 4b), which was further investigated using split luciferase complementation (SLC) assay (Fig. 5). Expectedly, StnCBP interacted with HC-Pro^O^ and HC-Pro^N^ (Fig. 5a and b). Moreover, with the equal expression of the fusion proteins as determined using Western blotting, the leaf spots co-expressing StnCBP-HA-NLuc and CLuc-GFP-HC-Pro^O^ showed stronger luminescent signals than those co-expressing StnCBP-HA-NLuc and CLuc-GFP-HC-Pro^N^ (Fig. 5c–e), suggesting that StnCBP has a stronger interaction with HC-Pro^O^ than with HC-Pro^N^.

An alignment of the eIF4E-binding motifs in both EXA1 orthologues and HC-Pro/VPg proteins of potyviruses demonstrated that tyrosine and leucine are two conserved residues in the eIF4E-binding motifs (see online supplementary data Fig. S9a). Therefore, we designed a mutation to substitute tyrosine and leucine with alanine in the eIF4E-binding motifs of StEXA1 and HC-Pro^O^ proteins (namely StEXA1^Y298A, L304A^ and HC-Pro^O/Y343A, L348A^). Subsequent Y2H and Co-IP assays revealed that both mutations abolished the interaction of StnCBP with StEXA1 and HC-Pro^O^ (Fig. 4c and d). These results indicate that the tyrosine and leucine in eIF4E-binding motifs of both StEXA1 and HC-Pro^O^ are vital for their interactions with StnCBP, which is consistent with the previous research [49, 58, 59].

Overall, the above results indicate that StEXA1 and HC-Pro of PVY directly interact with StnCBP protein depending on their eIF4E-binding motifs, and the different interaction strengths between StnCBP and the two HC-Pro proteins may be responsible for the diverse phenotypes of RiStEXA1 lines in response to various PVY strains.

### *StnCBP* knockdown also significantly compromised PVY^O^ accumulation but not PVY^N:O^ aand PVY^NTN^

**Figure 6:**
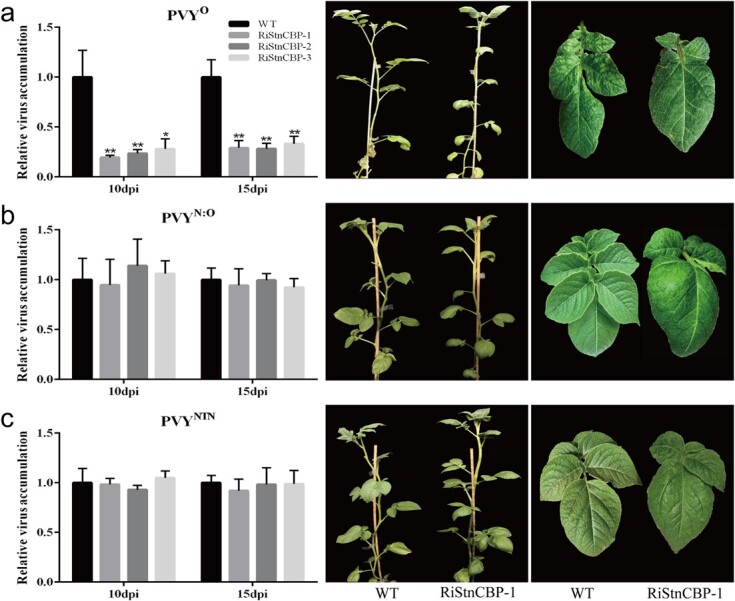
The viruses accumulate and symptoms develop in the RiStnCBP and control plants (WT) inoculated with PVY^O^ (**a**), PVY^N:O^ (**b**), and PVY^NTN^ (**c**). The relative virus accumulation was determined using qRT–PCR with total RNAs extracted from the non-inoculated upper leaves at 10 and 15 dpi, respectively. Symptoms on plants and systemic leaves were observed and photos were taken at ~25 dpi. Data are presented as means ± SD (*n* = 3) relative to WT plants, and *EF1α* was used as the normalizer. Three independent experiments were performed with similar results. Asterisks indicate statistically significant differences according to Student’s *t* test (^**^P < 0.01, ^*^P < 0.05).

To confirm that StnCBP participates in PVY^O^ infection by associating with StEXA1 and HC-Pro^O^, we generated several RNAi transgenic lines of *StnCBP*. *StnCBP* knockdown seemed to interfere with the development of compound leaves, resulting in abnormal and deformed young leaves in potato, but had no obvious effect on the plant growth (Fig. 6). Three transgenic lines with high interference efficiency (RiStnCBP-1, RiStnCBP-2, and RiStnCBP-3) (see online supplementary data Fig. S4b) were selected and inoculated with PVY^O^, PVY^N:O^, and PVY^NTN^. The results were consistent with those obtained with the RiStEXA1 lines. *StnCBP* knockdown only compromised PVY^O^ accumulation but not the other tested viruses (Fig. 6 and online supplementary data Fig. S6d). Therefore, *StnCBP* and *StEXA1* possibly share the same pathway, which is important for PVY^O^ accumulation in potato.

### Distinct spatial conformations of HC-Pro^O^ and HC-Pro^N^ proteins contributed to their different interaction strengths with StnCBP

**Figure 7:**
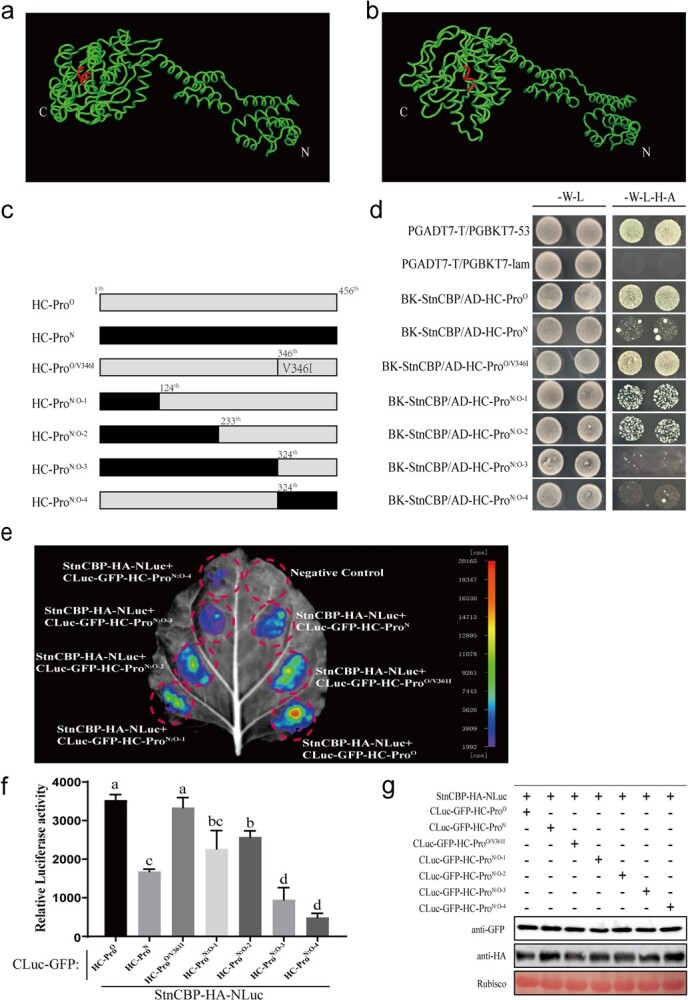
Distinct spatial conformations of HC-Pro^O^ and HC-Pro^N^ proteins contributed to their different interaction strengths with StnCBP. Three-dimensional structural models of HC-Pro^O^ (**a**) and HC-Pro^N^ (**b**) were predicted using ROBETTA. The red area indicates the eIF4E-binding motif. N and C indicate the N- and C-terminal of HC-Pro protein, respectively. **c** Schematic illustrations of mutational and recombinant HC-Pro proteins. Black and gray bars represent the sequences derived from HC-Pro^N^ and HC-Pro^O^, respectively. V346I denotes the substitution of 346^th^ valine to isoleucine. Locations at the amino acid sequences indicate the mutational site and recombinant joints in HC-Pro. **d** Interactions between StnCBP and HC-Pro mutants/recombinations in Y2H assays. -W-L represents medium lacking tryptophan and leucine, while -W-L-H-A represents medium lacking tryptophan, leucine, histidine, and adenine. Paired combinations PGADT7-T/PGBKT7–53 and PGADT7-T/PGBKT7-lam represent positive and negative controls, respectively. **e**, **f**, and **g** Interactions between StnCBP and HC-Pro mutants/recombinations in the SLC assays. The luminescence images were captured using a CCD imaging system and the pseudocolor bar shows the range of luminescence intensity. The quantification of relative luciferase activity in leaves is equivalent to luminescence intensity/50 mm^2^ leaf area, which was calculated using IndiGO imaging software. Each bar represents the mean ± SD of three replicates (*n* = 3). According to ordinary one-way ANOVA with multiple comparisons, different letters indicate significant differences at *P* < 0.05. Western blotting showed the protein abundance of NLuc and CLuc fusions.

Previous Y2H assay had demonstrated that StnCBP had strong and weak interaction with HC-Pro^O^ and HC-Pro^N^, respectively (Fig. 4b and Fig. 5c–e). The reasons for the difference in interaction strength were investigated using a sequence alignment analysis between HC-Pro^O^ and HC-Pro^N^. The results showed that they shared a 91% homology at the protein level (see online supplementary data Fig. S9b). Different amino acid residues were discretely distributed on the full-length protein, including the eIF4E-binding motifs of HC-Pro^O^ (YINVFLA) and HC-Pro^N^ (YINIFLA). An artificial mutant with the substitution of valine by isoleucine in the eIF4E-binding motif of HC-Pro^O^ (named HC-Pro^O/V346I^) was constructed to determine whether the difference in the eIF4E-binding motif affects the interaction strength (Fig. 7c). Subsequent Y2H and SLC assays demonstrated that HC-Pro^O/V346I^ still displayed a strong interaction with StnCBP (Fig. 7d–g), indicating that the difference in the eIF4E-binding motif between HC-Pro^O^ and HC-Pro^N^ does not affect the interaction.

Since the different amino acid residues between HC-Pro^O^ and HC-Pro^N^ were discretely distributed, the spatial conformation of the proteins might be responsible for the difference in interaction. Using ROBETTA (https://robetta.bakerlab.org/) and PyMOL (https://pymol.org/2/), the tertiary protein structures of HC-Pro^O^ and HC-Pro^N^ were predicted and visualized as shown in Fig. 7a and b. Obviously, the spatial conformations of HC-Pro^O^ and HC-Pro^N^ were similar in the N-terminal half but significantly different in the C-terminal half containing the eIF4E-binding motif.

To test whether the C-terminals of HC-Pro^O^ and HC-Pro^N^ were vital for their interaction with StnCBP, we constructed a series of artificial recombinants between HC-Pro^O^ and HC-Pro^N^ as follows: N-terminal one-quarter (~124 amino acids) of HC-Pro^N^ recombined with C-terminal three-quarters of HC-Pro^O^ (named as HC-Pro^N:O-1^), N-terminal half (~233 amino acids) of HC-Pro^N^ recombined with C-terminal half of HC-Pro^O^ (named as HC-Pro^N:O-2^), N-terminal three-quarters (~324 amino acids) of HC-Pro^N^ recombined with C-terminal one-quarter of HC-Pro^O^ (named as HC-Pro^N:O-3^), N-terminal three-quarters (~324 amino acids) of HC-Pro^O^ recombined with C-terminal one-quarter of HC-Pro^N^ (named as HC-Pro^N:O-4^) (Fig. 7c). Then, the Y2H and SLC assays showed that the recombination of HC-Pro^N:O-1^ and HC-Pro^N:O-2^ debilitated their interaction with StnCBP, while the recombination of HC-Pro^N:O-3^ and HC-Pro^N:O-4^ almost abolished their interaction with StnCBP (Fig. 7d–g). These results are consistent with the differences between the two HC-Pro proteins in spatial conformation. Obviously, the C-terminal half of the HC-Pro^O^, particularly the moiety near the eIF4E-binding motif, is essential for its interaction with StnCBP. Together with the mutant assay in the eIF4E-binding motif, it can be speculated that the spatial conformation near the eIF4E-binding motif is likely responsible for the different interaction strengths of StnCBP with HC-Pro^O^ and HC-Pro^N^.

### Stress granules were induced either by HC-Pro^N^ independently or by StEXA1 and StnCBP coordinately in *N. benthamiana*

**Figure 8:**
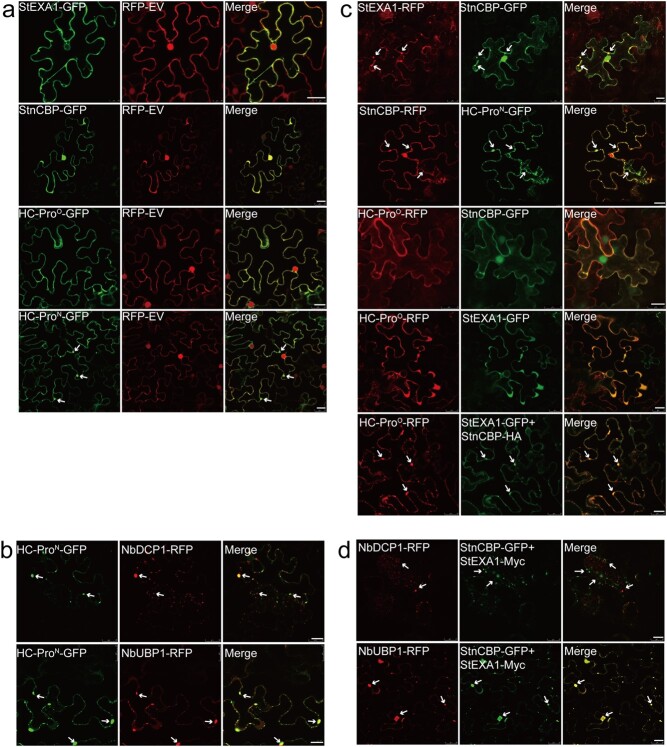
Subcellular localization and co-localization assays reveal that SGs can be either by HC-Pro^N^ independently or by StEXA1 and StnCBP coordinately in *N. benthamiana*. **a** Subcellular localization of StEXA1, StnCBP, HC-Pro^O^, and HC-Pro^N^. **b** Co-localization of HC-Pro^N^ with NbDCP1 and NbUBP1. **c** Co-localization between StEXA1, StnCBP, HC-Pro^O^, and HC-Pro^N^. **d** Co-localization of StEXA1/StnCBP with NbDCP1 and NbUBP1. The fluorescence signals were observed using a confocal microscope two days after agroinfiltration in *N. benthamiana* leaves. RFP-EV indicates an empty RFP vector (pK7WGR2) as the control. Merge means the overlay of GFP and RFP on single confocal planes. Arrows indicate granule-like structures. Scale bar: 20 μm.

To analyse where StEXA1, StnCBP, and HC-Pro may interact and function *in vivo*, we observed their subcellular localizations in *N. benthamiana* leaves. The results revealed that StnCBP was localized to both cytoplasm and nucleus while StEXA1, HC-Pro^O^, and HC-Pro^N^ were mainly localized to the cytoplasm (Fig. 8a), which is consistent with previous studies [61, 62]. Interestingly, some cytoplasmic granule-like structures were observed in the subcellular localization of HC-Pro^N^, but not in that of HC-Pro^O^, StEXA1, or StnCBP (Fig. 8a). In Eukaryota, mRNA is assembled into cytoplasmic messenger ribonucleoprotein (mRNP) complexes, which then form large RNA granules from either translation initiation complexes (called stress granules, SGs) or mRNPs in the degradation state (called processing bodies, PBs) [63, 64]. SGs and PBs are the two most well-known RNA granules, and increasing evidence indicates that they interplay with viruses to either support their infection cycle or participate in the antiviral defense in both animal and plant kingdoms [65, 66]. Two marker proteins, NbUBP1 (oligouridylate binding protein 1, UBP1) and NbDCP1 (decapping protein 1), respectively specific for SGs and PBs [66], were used in a co-localization assay to determine whether the RNA granules induced by HC-Pro^N^ in this study are SGs or PBs. As a result, NbDCP1 was partially co-localized while NbUBP1 was fully co-localized with HC-Pro^N^ (Fig. 8b), suggesting that the granules observed in the subcellular localization of HC-Pro^N^ were most likely SGs. Similar results were obtained in previous research on PVA, which demonstrated that the HC-Pro of PVA induces the assembly of RNA granules (referred to as noncanonical SGs or potyvirus-induced granules (PGs)). These granules were found to possess UBP1, HC-Pro, VPg, eIF(iso)4E, acidic ribosomal protein P0, argonaute 1 (AGO1), and many other host factors, and thus considered to be associated with viral translation and suppression of antiviral RNA silencing [67, 68].

Then, co-localization assays of StEXA1, StnCBP, HC-Pro^O^, and HC-Pro^N^ were further performed (Fig 8c). When the paired interacting proteins (StnCBP/StEXA1, StnCBP/HC-Pro^O^, and StnCBP/HC-Pro^N^) were co-expressed, the fluorescence signals of GFP and RFP were all mainly co-localized in the cytoplasm (Fig. 8c). These results agreed with those of the Y2H and Co-IP assays, suggesting that their interactions occur in the cytoplasm. Expectedly, HC-Pro^N^ and StnCBP were co-localized in cytoplasmic granule-like structures because HC-Pro^N^ itself could induce the assembly of SGs. Surprisingly, cytoplasmic granule-like structures were also observed in the co-localization of StEXA1 and StnCBP. Moreover, when HC-Pro^O^ was co-expressed with StEXA1 and StnCBP, co-localization signals were also observed in the granules in the cytoplasm, but co-expression of either StEXA1 or StnCBP with HC-Pro^O^ induced no granule-like structure (Fig. 8c). Both EXA1 and nCBP in *Arabidopsis* were found to be components of the 5′ cap complex required for translation of mRNAs in a cap-dependent manner [69]. Thus, the granules induced by StEXA1 and StnCBP were probably SGs. To test this possibility, StEXA1 and StnCBP together were co-localized with the marker protein NbDCP1 or NbUBP1. Obviously, NbDCP1 was hardly co-localized while NbUBP1 was completely co-localized with the granules induced by StEXA1 and StnCBP (Fig. 8d), which also suggested that the granules were SGs. These results indicated that HC-Pro^O^ participates in the SG-dependent RNA regulatory process by manipulating StEXA1 and StnCBP, while HC-Pro^N^ is independently involved in this process through some unknown mechanism.

## Discussion

In addition to *eIF4Es*, increasing susceptibility genes have been found to be vital for the viral infection cycle and are considered potential recessive resistance genes and promising targets in the breeding of crops [19, 20]. Recessive resistance conferred by critical susceptibility genes or host factors is more broad-spectrum and durable against virial pathogens compared with dominant resistance conferred by *R* genes [18]. For example, loss-of-function mutation of eIF4Es would result in broad-spectrum resistance to potyviruses, potexviruses, bymoviruses, cucumoviruses, ipomoviruses, and carmoviruses [70–75]. *EXA1* has been reported as a recessive resistance gene required for the infection of viruses, bacteria, and oomycete pathogens in several model plants [44–47]. Noteworthy, EXA1-mediated resistance is broad-spectrum for multiple members of the genus *Potexvirus* and conserved in *A. thaliana*, tobacco, and tomato [44, 45]. In this study, *StEXA1* was cloned in potato, whose function in response to several potato viruses was further investigated in RNAi transgenic plants. Unexpectedly, *StEXA1* knockdown did not interfere with PVX infection (Fig. 1), which is inconsistent with the previous finding that *NbEXA1* knockdown compromised the accumulation of two potexviruses including PVX, even though the gene silencing efficiency of *EXA1* was similar in the two species [45]. Moreover, a ~38% knockdown of *SlEXA1* in tomato also suppressed the accumulation of pepino mosaic virus (PepMV, potexvirus) [45]. These results together indicate that partial silencing of *EXA1* may result in a different response of potato to potexviruses compared with tobacco and tomato, and knockout of *StEXA1* may need be carried out in the future to further confirm this different response. In addition, *StEXA1* is associated with the susceptibility to PVY, and *StEXA1* knockdown in potato significantly reduced PVY^O^ accumulation. To the best of our knowledge, this is the first report that *EXA1* acts as a recessive resistance gene against a potyvirus, which can improve the understanding of *EXA1* function. Moreover, the *StEXA1-*mediated resistance seems to be strain-specific in response to different PVY strains: it effectively suppressed PVY^O^ accumulation but not PVY^N:O^ and PVY^NTN^ (Fig. 2), indicating that the function of *StEXA1* is dependent on its recognition of the right viral protein.

Through the construction and modification of an infectious clone of PVY^O^, HC-Pro was identified as the vital protein involved in the *StEXA1*-mediated recessive resistance against PVY^O^ (Fig. 3). However, StEXA1 did not interact with HC-Pro or any other proteins of PVY (see online supplementary data Fig. S8a), suggesting that some other host factors might be involved in the recognition of HC-Pro in *StEXA1*-mediated recessive resistance.

Based on the two conserved domains (eIF4E-binding motif and GYF domain), several hypotheses have been proposed to explain the possible molecular function of EXA1. The first hypothesis is that EXA1 acts as a component of the translation initiation factor complex to control mRNA (or virus RNA similar to mRNA) translation via interaction with eIF4Es [44]. This hypothesis is buttressed by the finding that an *ncbp* mutant in the eIF4E family had a lower accumulation of three potexviruses than the *exa1–1* mutant did in *Arabidopsis* [44, 71]. In addition, AtEXA1 was found to be involved in translation regulation by interacting with a ribosomal protein RPL18 and two eIF4E initiation factors eIF4E1 and eIF4E1B [47]. The second hypothesis is that EXA1 binds to the PRS of other factors via the GYF domain and assists the function of certain viral factors [44]. Unfortunately, studies and experimental data are insufficient, and such RPS-containing host factors interacting with the GYF domain of *EXA1* remain unidentified. The final hypothesis suggests that EXA1 works as a translational repressor, which negatively regulates the accumulation of nucleotide-binding leucine-rich repeat proteins (NLRs) and reduces general disease resistance. In the *exa1–1* mutant, the protein levels of several NLRs significantly increased and the mutant plants exhibited enhanced resistance to bacterial and oomycete pathogens [47].

This study provides more powerful evidence to support the first hypothesis. StEXA1 directly interacts with at least two members of eIF4Es in potato, one of which (StnCBP) can interact with the HC-Pro protein, a putative key PVY protein in *StEXA1-*mediated resistance to PVY^O^. Knockdown of either *StEXA1* or *StnCBP* suppressed PVY^O^ accumulation but not PVY^N:O^ and PVY^NTN^. These results indicate a link between EXA1 and viral translation depending on eIF4Es. Several studies in various plants have suggested that the interactions between eIF4Es and VPgs are responsible for *eIF4E*-mediated resistance [76–79]. However, the interaction between eIF4Es and VPg/NIa alone cannot fully elucidate the role of eIF4Es in the infection cycle of potyviruses. Moreover, the HC-Pro of PVA interacts with eIF4E and eIF(iso)4E depending on the eIF4E-binding motif, which is important for viral virulence [58]. The HC-Pro of peanut stripe virus (PStV) was also shown to interact with PeaeIF4E and PeaeIF(iso)4E in the cytoplasm [26]. In this study, the HC-Pro of PVY interacted with the eIF4E-type protein StnCBP in both Y2H and Co-IP assays, while the VPg of PVY and StnCBP proteins showed no interaction signal in the Y2H assay (see online supplementary data Fig. S8b). In a recent study of sugarcane, the interactions between ScnCBP and VPgs of several sugarcane mosaic pathogens were detected only in bimolecular fluorescence complementation (BiFC), but not in Y2H assay [79]. Thus, we cannot exclude the possibility that the VPg or some other proteins of PVY may also be involved in the resistance mediated by StEXA1 and StnCBP. Moreover, further research is needed to investigate the interactive relationship between StEXA1/StnCBP and the proteins of PVX, which may explain why the knockdown of *StEXA1* and *StnCBP* did not influence PVX accumulation.

**Figure 9:**
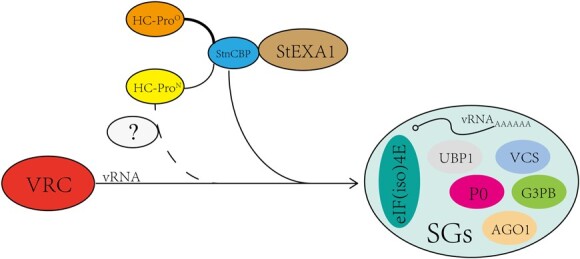
A hypothetical working model for StEXA1 and StnCBP as susceptibility factors associated with PVY infection in potato. StEXA1 and StnCBP interact and participate in the assembly of SGs as potential SGs components. PVY^O^ may manipulate the StEXA1/StnCBP complex to help the viral RNA (vRNA) penetrate the SGs to facilitate virus accumulation through the interactions between HC-Pro^O^ and StnCBP. Conversely, HC-Pro^N^ of PVY^N^ and PVY^NTN^ itself can induce the formation of SGs through an unknown mechanism. However, it may also be involved in the same pathway depending on StEXA1 and StnCBP through a weaker interaction between HC-Pro^N^ and StnCBP. This model explains that the phenotypes knockdown of StEXA1 or StnCBP compromises PVY^O^ accumulation but not PVY^N:O^ and PVY^NTN^. SGs: stress granules; VRC: virus replication complex; vRNA: viral RNA.

The co-localization assays of the paired interacting proteins revealed that StEXA1/StnCBP is associated with the RNA regulatory process involving SGs. By combining all the above results, we propose a hypothetical working model for the association of StEXA1 and StnCBP with PVY accumulation in potato (Fig. 9). Both StEXA1 and StnCBP act as susceptibility factors, and can interact with each other to participate in the assembly of SGs as potential SG components. Depending on the interaction between HC-Pro^O^ and StnCBP, PVY^O^ may manipulate the StEXA1/StnCBP complex to help the viral RNA (vRNA) penetrate the SGs to facilitate virus accumulation. Conversely, HC-Pro^N^ of PVY^N:O/NTN^ can induce the formation of SGs through an unknown mechanism involving other host factors independently, or through the weaker interaction between HC-Pro^N^ and StnCBP. Therefore, knockdown of *StEXA1* or *StnCBP* prevents PVY^O^ but not PVY^N:O^ and PVY^NTN^ from hijacking the SG-dependent RNA regulatory pathway, thus compromising the accumulation of the virus. Our findings provide new insights into the function of StEXA1 and the underlying mechanism involving the host RNA regulatory network.

## Materials and methods

### Plant growth conditions and virus isolates

Virus-free tissue culture plantlets of potato cultivar Eshu 3, and transgenic lines were maintained *in vitro* (20°C, 16 h of light/8 h of dark, 400–1000 μmol photons m^−2^ s^−1^) on an MS medium supplemented with 4% sucrose [80]. For resistance assays, the plantlets were transplanted into pots (12 cm) containing premixed soil in the greenhouse (18–22°C, photoperiod of 12 h/d, 90 μmol photons m^−2^ s^−1^) at Huazhong Agricultural University (HZAU).

Five virus isolates were used in this study. PVX-HB3 and PVM-HB36 isolates were collected and isolated from a local potato virus survey, and three PVY strains/isolates (PVY^O^-FL, PVY^N:O^-Mb58, and PVY^NTN^-S1) were from Agriculture and Agri-Food Canada’s Fredericton Research and Development Centre [81]. These viruses were maintained in tobacco or potato host plants in the greenhouse at HZAU. Before inoculation, the viral identity and purity were verified as described previously [81, 82].

### BLAST search, cloning, and phylogenetic analysis

A BLASTp search was performed using the AtEXA1 (AT5G42950) amino acid sequence as a query against the potato reference genome *S. tuberosum* group Phureja DM1–3 v6.1 (Spud DB, http://potato.plantbiology.msu.edu/) to retrieve the putative orthologous protein in potato (StEXA1). The potential interacting proteins of StEXA1 were predicted in the STRING database (https://www.string-db.org/) and retrieved from Spud DB using the resulting gene IDs. Then, these genes were amplified with corresponding gene-specific primers (see online supplementary data Table S3) from the potato cultivar Eshu 3 using Phanta Super-Fidelity DNA polymerase (Vazyme, Nanjing, China), and the PCR amplicons were cloned into the pCE-Zero vector (Vazyme, Nanjing, China). At least five colonies for each target gene were randomly selected and sent for sequencing (Sangon Biotech Co., Ltd, Shanghai, China). The alignment of gene nucleotide sequences from Eshu 3 and Phureja DM1–3 was performed by DNAMAN. For phylogenetic analysis, BLASTp searches against *S. tuberosum* group Phureja DM1–3 v6.1 (Spud DB), *N. benthamiana* Genome v1.0.1 (*Solanaceae* Genomics Network, SGN, https://solgenomics.net/) and Tomato Genome proteins (ITAG release 4.0) (SGN) were performed using the amino acid sequences of EXA1 and GYF domain containing proteins in *A. thaliana* [44] as queries to obtain their putative orthologues in *S. tuberosum, N. benthamiana* and *S. lycopersicum*, respectively*.* Then, a phylogenetic tree was generated with Neighbor-Joining Tree and 1000 bootstraps by MEGA5.2.

### RNA extraction, RT-PCR, and RT-qPCR

Total RNA was extracted from fresh leaf samples using the Total RNApure Kit (ZOMANBIO, Beijing, China). The first-stand cDNA was synthesized using TRUE RT Master Mix (AidLab Biotech, Beijing, China) following the manufacturer’s instructions. PCR was conducted using appropriate primers (Supplementary data Table S3) and Phanta Max Master Mix (Vazyme, Nanjing, China) on a C1000 Thermal Cycler (Bio-rad Laboratories, Hercules, USA), while quantitative PCR was performed using Bio-Rad CFX96™ Real-time System (Bio-rad Laboratories) with 2× qPCR Real-Time Kit (Applied Biological Materials (abm) Inc., Vancouver, Canada). The 2^-ΔΔCq^ method was used for gene expression level analysis based on potato internal reference gene *elongation factor 1 alpha* (*EF1α*, accession number: Soltu.DM.06G005620) in RT-qPCR [83]. All histograms were made using GraphPad Prism.

### Potato transformation

For knockdown of *StEXA1* and *StnCBP* expression, Eshu 3 was selected as the receptor for genetic transformation, and the target fragments for RNAi were designed based on the nucleotide sequence of *StEXA1* and *StnCBP* cloned from Eshu 3. Then the fragments were amplified and inserted into the *XhoI* and *XbaI* sites of the pHellsGate8 vector using the ClonExpress II One Step Cloning Kit (Vazyme, Nanjing, China). Vectors were electroporated into *Agrobacterium tumefaciens* (*A. tumefaciens*), GV3101, and then transformed into microtuber slices of Eshu 3 as previously described [84]. Transformants were screened via secondary rooting on a selective medium with Kanamycin, and the interference efficiency of the corresponding gene was evaluated using RT-qPCR. The transgenic line was named as gene names with a prefix ‘Ri’ representing RNA interference and followed by a number representing the serial number of the transformant. Primers for constructing RNAi vectors are shown in online  supplementary data Table S3.

### Construction of PVY infectious cDNA clone

To construct PVY^O^ infectious cDNA clone, we used homologous recombination in yeast to assemble the full-length PVY^O^ infectious clone (named pCB301-2μ: PVY^O^) into pCB301-2μ-HDV using six overlapping DNA fragments, including four fragments from PVY^O^ (PVY-A, PVY-B1, PVY-B2, and PVY-C), a linearized plasmid between the CaMV 35S promoter and the HDRz sequence of pCB301-2μ-HDV produced using PCR, and Intron2 from bean *NIR* gene (accession number: U10419.1). The Intron2 was inserted into the CI protein to abolish the toxicity to *Escherichia coli*. The detailed steps are similar as described previously [85]. To obtain pCB301-2μ: PVY^O^ with HC-Pro^N^, the plasmid pCB301-2μ: PVY^O^ was linearized between the P1 and P3 sequences using PCR. Then, the HC-Pro^N^ fragment was assembled using ClonExpress II One-Step Cloning Kit (Vazyme, Nanjing, China). The infectious cDNAs of viruses were stored in tobacco via Agrobacterium infiltration. Primers for constructing pCB301-2μ: PVY^O^ and pCB301-2μ: PVY^O/HC-ProN^ are shown in Supplementary data Table S4.

### Virus inoculation and resistance assays

For viral inoculation, at least six potato plants at the 4–6 leaf stage were inoculated with each viral inocula (leaf extract: approximately 1 g leaf tissue homogenized in 10 ml 10-mM phosphate buffer, pH 7.5, with 32 mM sodium sulfite) via mechanical wounding as described previously [86]. Foliage symptoms were monitored daily after inoculation until harvest.

For resistance assays, upper non-inoculated leaves from inoculated plants were collected at 10 and 15 dpi for ELISA assays using virus-specific antibodies (Agdia, Elkhart, IN, USA) following the manufacturer’s guidelines as previously described [87].

### Yeast two-hybrid assays

For yeast two-hybrid (Y2H) assays, *StEXA1*, *StEXA1*^Y298A, L304A^, HC-Pro^O^, HC-Pro^N^, HC-Pro^O/Y343A, L348A^, and five recombinant HC-Pro proteins were inserted between the *BamHI* and *EcoRI* sites of pGADT7, SteIF4E, SteIF(iso)4E, StnCBP, while 11 PVY^O^ proteins were inserted between the *BamHI* and *EcoRI* sites of pGBKT7 using SE cloning kit (Applied Biological Materials (abm) Inc., Vancouver, Canada). Paired combinations for interaction analysis were co-transformed into *Saccharomyces cerevisiae* strain, AH109. Positive transformants were selected via a medium lacking leucine and tryptophan. Then, the interactions were identified using a medium that contains X-α-GAL (20 mg/L) and lacks leucine, tryptophan, adenine, and histidine. Primers for constructing Y2H vectors are shown in online supplementary data Table S5.

### Split luciferase complementation (SLC) assay

The detailed protocol was referred to in a previous study [88]. Briefly, paired combinations for interaction analysis were inserted into NLuc and CLuc plasmids. The two plasmids were added with HA-tag and GFP-tag, respectively, to facilitate the detection of protein expression using Western blotting. Then, *Agrobacterium* containing the two plasmids was infiltrated into *N. benthamiana* leaves at the 4–6 stage. The concentrations of *Agrobacterium* were measured and uniformly resuspended to OD600 = 0.5 to ensure consistent protein expression levels. Finally, the luminescence images were captured using NightSHADE LB 985 (Berthold Technologies, Germany) at 48 h after infiltration. The relative luciferase activity is equivalent to luminescence intensity/50 mm^2^ leaf area, and the protein expression levels were detected using Western blotting. Primers for constructing SLC vectors are shown in Supplementary data Table S6.

### Subcellular localization

To observe the subcellular localization of StEXA1, StnCBP, HC-Pro^O^, and HC-Pro^N^, corresponding fragments amplified by specific primers (see online supplementary data Table S4) were inserted into the *Bsp1407I* site of pK7WGF2 and fused behind eGFP. Additionally, *StEXA1*, *StnCBP*, *NbDCP1* (accession number: Niben101Scf08515g00023), and *NbUBP1* (accession number: Niben101Scf08651g00012.1) were inserted into the *Bsp1407I* site of pK7WGR2 and fused behind RFP to achieve co-localized analyses. The vectors were electroporated into *A. tumefaciens*, GV3101, and injected into the leaves of *N. benthamiana* at the 4–6 leaf stage. GFP fluorescence was observed at 48 h after injection using a confocal laser scanning microscope (SP8, Leica, Wetzlar, Germany). Primers for constructing subcellular localization vectors are shown in Supplementary data Table S6.

### Co-immunoprecipitation and Western blot assay

*StEXA1*, *HC-Pro^O^*, and *HC-Pro^N^* were amplified and inserted into the *StuI* site of pH 7LIC9.0-N-Myc, while *StnCBP* was amplified and inserted into pH 7LIC7.0-N-HA for Co-Immunoprecipitation (Co-IP) assays. Paired combinations were co-expressed in the leaves of *N. benthamiana* at the 4–6 leaf stage. The samples were harvested at 48 h post-infection and the proteins were extracted using a protein extraction buffer [100-mM Tris-HCl, pH 8.0, 5-mM EDTA, 150-mM NaCl, 10% glycerol, 2 -mM dithiothreitol (DTT), 1% protease inhibitor tablets (A32955; ThermoFisher Scientific), 2-mM phenylmethylsulfonyl fluoride (PMSF)]. Then, 15-μl beads (Anti-Myc mAb-Magnetic beads, D153-10, MBL, Tokyo, Japan) were added to 500-μl extracted protein supernatant. After incubation at 4°C for 2 hours, the beads were washed thrice with wash buffer (100-mM Tris-HCl, pH 8.0, 5-mM EDTA, 150-mM NaCl, 10% glycerol, 2-mM PMSF). Finally, the beads were added with 50-μl 5× SDS buffer containing 5% β-mercaptoethanol, and boiled at 95°C for 10 minutes to elute the proteins for Western blot assay as previously described [60]. Primers for constructing Co-IP vectors are shown in online  supplementary data Table S6.

### Statistical analyses

All data are expressed as the mean value ± standard deviation (SD) of biological replicates. Statistical significance was determined using Student’s t-test.

## Supplementary Material

Web_Material_uhac159

## Data Availability

All data supporting the findings of this research are available within the paper and within its online supplementary data. Accession numbers: AT5G42950 (*A. thaliana* EXA1), Soltu.DM.04G035210 (*S. tuberosum* EXA1), Solyc04g080240 (*S. lycopersicum* EXA1), Niben101Scf04831g00010 (*N. benthamiana* EXA1), Soltu.DM.10G026730 (*S. tuberosum* nCBP), Soltu.DM.03G000970 (*S. tuberosum* eIF4E), Soltu.DM.09G027260 (*S. tuberosum* eIF(iso)4E), Soltu.DM.06G005620 (*S. tuberosum* EF1α), U10419.1 (*Phaseolus vulgaris NIR*), Niben101Scf08515g00023 (*N. benthamiana* DCP1), and Niben101Scf08651g00012 (*N. benthamiana* UBP1).
