# Supplementary material for: Susceptibility factor StEXA1 interacts with StnCBP to facilitate potato virus Y accumulation through the stress granule-dependent RNA regulatory pathway in potato
Source: Hortic Res. 2022 Jul 22;9:uhac159. doi: 10.1093/hr/uhac159 (PMC9531334; doi:10.1093/hr/uhac159)
Supplement: Web_Material_uhac159 [file web_material_uhac159.zip › supplementary information 6.27.docx]

**Supplementary information**

**Table S3. Primers for gene cloning, constructing RNAi vectors and RT-qPCR.**

| **Primer name** | **Sequence (5'-3')** | | **Note** |
| --- | --- | --- | --- |
| StEXA1-F | | ATGGGTGACAAAACTCAATTTGATTCT | to amplify full-length cDNA of *StEXA1* |
| StEXA1-R | | CTAGTCTTCCACAGTCTGAATCTCTC |  |
| StnCBP-F | | ATGGAAGTGACACCGGAGAAGA | to amplify full-length cDNA of *StnCBP* |
| StnCBP-R | | TTATGATCTTATCCAAGTATTGCGGTAAG |  |
| StEXA1-XhoIF | | TTTGGAGAGGACACGCTCGAGGATGTGCAAGGACCCAACGA | to construct RNAi vector of *StEXA1* |
| StEXA1-XhoIR | | TGGGGTACCGAATTCCTCGAGCTTGCGGCCATCACCAATCT |  |
| StEXA1-XbaIF | | TCATTAAAGCAGGACTCTAGAGATGTGCAAGGACCCAACGA | to construct RNAi vector of *StEXA1* |
| StEXA1-XbaIR | | GATAAGCTTGGATCCTCTAGACTTGCGGCCATCACCAATCT |  |
| StnCBP-XhoIF | | TTTGGAGAGGACACGCTCGAGCTCAATAGCCGCCGAGGATC | to construct RNAi vector of *StnCBP* |
| StnCBP-XhoIR | | TGGGGTACCGAATTCCTCGAGCCCTCTTTGAAAAGATGCAAATCAG |  |
| StnCBP-XhoIF | | TCATTAAAGCAGGACTCTAGACTCAATAGCCGCCGAGGATC | to construct RNAi vector of *StnCBP* |
| StnCBP-XbaIR | | GATAAGCTTGGATCCTCTAGACCCTCTTTGAAAAGATGCAAATCAG |  |
| EF1α-F | | ATTGGAAACGGATATGCTCCA | reference gene for real time PCR |
| EF1α-R | | TCCTTACCTGAACGCCTGTCA |  |
| StEXA1-QF | | GATCTACGTCTCACGGCAGG | primers for real time PCR |
| StEXA1-QR | | TTATCATGGGGCTCCCTCCA |  |
| StnCBP-QF | | TCGTAATGCGTCAGATCAGCAGG | primers for real time PCR |
| StnCBP-QR | | GCGCAAGGAAGCATCATGGG |  |
| PVY-QF | | ACACCAGTGAGGGCTAGGGA | primers for real time PCR |
| PVY-QR | | GTGGTGTGCCTCTCTGTGTT |  |
| PVX-QF | | GTACAGCTCGTGCCATAGTAG | primers for real time PCR |
| PVX-QR | | TGTGGGCACCTTCATGTCCTTC |  |
| PVA-QF | | CATGCTTGACCATGCAAAGCC | primers for real time PCR |
| PVA-QR | | CTCTCAGGTTGCGTTGAAGACC |  |
| PVM-QF | | CGAAGGGTGTGTAGGCTGTATG | primers for real time PCR |
| PVM-QR | | CAGCGAAGCGATCCTCATACT |  |

**Table S4. Primers for constructing pCB301-2μ: PVY^O^ and pCB301-2μ: PVY^O/HC-ProN^.**

| **Primer name** | **Sequence (5'-3')** | **Note** |
| --- | --- | --- |
| PVY-A-JF | CATTTCATTTGGAGAGGAATTAAAACAACTCAATACAACATAAG | to amplify fragment of PVY-A |
| PVY-A-JR | TATTGCCTGACACACTGCCA |  |
| PVY-B1-JF | ATCTTTAGGCGTTTGCCAAC | to amplify fragment of PVY-B1 |
| PVY-B1-JR | TAAGTGCATACTTACAATCAGACTAGTTGAGACGCCT |  |
| Intron2-JF | TCAACTAGTCTGATTGTAAGTATGCACTTAAAGAGTATGTGTGG | to amplify fragment of intron2 |
| Intron2-JR | CACAGTACAATTGCCCTGCACAATTTCAAAGATTGAACCT |  |
| PVY-B2-JF | TTTGAAATTGTGCAGGGCAATTGTACTGTGCGCCA | to amplify fragment of PVY-B2 |
| PVY-B2-JR | ACAGGGAAATCTTTCGGCATTT |  |
| PVY-C-JF | TTGAGCGTTCTGCCAATTAAAGGT | to amplify fragment of PVY-C |
| PVY-C-JR | GTTTGAACGATCGGGGAAATTCTTTTTTTTTTTTTTTGTCTCCTGATTGAAGTT |  |
| PVY-backbone/F | AACTTCAATCAGGAGACAAAAAAAAAAAAAAAGAATTTCCCCGATCGTTCAAA | to linearize plasmid pCB301-2μ-HDV |
| PVY-backbone/R | CTTATGTTGTATTGAGTTGTTTTAATTCCTCTCCAAATGAAATGAACTTCCT |  |
| PVYO/NHC-backboneF | GGTGTTCCTAATGCATGCCCTG | to linearize plasmid pCB301-2μ-PVY^O^ |
| PVYO/NHC-backboneR | AAACTGGATCATTGAGTTCAAAACACTCT |  |
| NHC/PVYO-F | AACTCAATGATCCAGTTTTCAAGCGCTGAAAGCTTTTGGA | to amplify fragment of HC-Pro^N^ |
| NHC/PVYO-R | GCATGCATTAGGAACACCACCAACTCTATAGTGCTTAATGTCAGACT |  |

**Table S5. Primers for constructing Y2H vectors.**

| **Primer name** | **Sequence (5'-3')** | **Note** |
| --- | --- | --- |
| StEXA1-ADF | ATGGAGGCCAGTGAATTCATGGGTGACAAAACTCAATTTGATTCT | to construct vector pGADT7: StEXA1 |
| StEXA1-ADR | GCTCGAGCTCGATGGATCCCTAGTCTTCCACAGTCTGAATCTCTC |  |
| P1-BKF | GGCCATGGAGGCCGAATTCATGGCAACTTACATGTCAACAATCT | to construct vector pGBKT7: P1 |
| P1-BKR | CTGCAGGTCGACGGATCCTCAACTCTGAGTAACTCTAGAACGTGCA |  |
| HC-BKF | GGCCATGGAGGCCGAATTCATGTCGAATGCTGATAATTTTTGGAAGG | to construct vector pGBKT7: HC-Pro |
| HC-BKR | CTGCAGGTCGACGGATCCTCAACCAACTCTATAATGTTTTATATCAGA |  |
| P3-BKF | GGCCATGGAGGCCGAATTCATGGGTGTTCCTAATGCATGCCC | to construct vector pGBKT7: P3 |
| P3-BKR | CTGCAGGTCGACGGATCCTCACTGGTGTCGCACATCATATTCTT |  |
| PIPO-BKF | GGCCATGGAGGCCGAATTCATGGGAAAAAAATTATCTAAATCTCTTGAACGA | to construct vector pGBKT7: PIPO |
| PIPO-BKR | CTGCAGGTCGACGGATCCTCATCACTCAATCCTGAGGCAGTACC |  |
| 6K1-BKF | GGCCATGGAGGCCGAATTCATGCGTTCCACACCAGGTGTTAA | to construct vector pGBKT7: 6K1 |
| 6K1-BKR | CTGCAGGTCGACGGATCCTCACTGATGTCTAACTTCATAGTCCATT |  |
| CI-BKF | GGCCATGGAGGCCGAATTCATGTCCTTAGACGATGTGATCAAGAATT | to construct vector pGBKT7: CI |
| CI-BKR | CTGCAGGTCGACGGATCCTCATTGGTGATGAACGAACTGCAAAG |  |
| 6K2-BKF | GGCCATGGAGGCCGAATTCATGGCTGCGACGTCACTTGCAAA | to construct vector pGBKT7: 6K2 |
| 6K2-BKR | CTGCAGGTCGACGGATCCTCATTGGTGAGCCACAGTCTCAACTGAT |  |
| Vpg-BKF | GGCCATGGAGGCCGAATTCATGGGGAAAAATAAATCCAAAAGAATTC | to construct vector pGBKT7: Vpg |
| Vpg-BKR | CTGCAGGTCGACGGATCCTCATTCATGCTCCACCTCCTGTGCTG |  |
| NIa-BKF | GGCCATGGAGGCCGAATTCATGGCTAAATCGCTCATGAGAGGC | to construct vector pGBKT7: NIa |
| NIa-BKR | CTGCAGGTCGACGGATCCTCATTGCTCCACCACTACATCATGATCG |  |
| NIb-BKF | GGCCATGGAGGCCGAATTCATGGCTAAGCACTCTGCGTGGAT | to construct vector pGBKT7: Nib |
| NIb-BKR | CTGCAGGTCGACGGATCCTCATTGATGGTGTACTTCATAAGAGTC |  |
| CP-BKF | GGCCATGGAGGCCGAATTCATGGCAAATGACACAATCGATGCAGGAG | to construct vector pGBKT7: CP |
| CP-BKR | CTGCAGGTCGACGGATCCTCACATGTTCTTGACTCCAAGTAGAG |  |
| SteIF4E-BKF | GGCCATGGAGGCCGAATTCATGGCAACAGCTGAAATGGAGA | to construct vector pGBKT7: SteIF4E |
| SteIF4E-BKR | CTGCAGGTCGACGGATCCCTATACGGTGTAACGATTCTTGGCA |  |
| SteIF(iso)4E-BKF | GGCCATGGAGGCCGAATTCATGGCCACCGAAGCACCG | to construct vector pGBKT7: SteIF(iso)4E |
| SteIF(iso)4E-BKR | CTGCAGGTCGACGGATCCTCACACAGTATATCGGCTCTTAGCT |  |
| StnCBP-BKF | GGCCATGGAGGCCGAATTCATGGAAGTGACACCGGAGAAGA | to construct vector pGBKT7: StnCBP |
| StnCBP-BKR | CTGCAGGTCGACGGATCCTTATGATCTTATCCAAGTATTGCGGTAAG |  |
| HC/O-ADF | ATGGAGGCCAGTGAATTCATGTCGAATGCTGATAATTTTTGGAAGG | to construct vector pGADT7: HC-Pro^O^ |
| HC/O-ADR | GCTCGAGCTCGATGGATCCTCAACCAACTCTATAATGTTTTATATCAGA |  |
| HC/N-ADF | ATGGAGGCCAGTGAATTCATGTCAAGCGCTGAAAGCTTTTGGA | to construct vector pGADT7: HC-Pro^N^ |
| HC/N-ADR | GCTCGAGCTCGATGGATCCTCAACCAACTCTATAGTGCTTAATGTCAGAC |  |
| StEXA1/YALA-ADR | GCCATTTTTATCCGACTGGCTTGTATAGGAGAGGAAACATTTTCAGCT | to construct vector pGADT7: StEXA1^Y298AL304A^ |
| StEXA1/YALA -ADF | GCCAGTCGGATAAAAATGGCTGATGTGTATAGAGTTACAGACATGCAG |  |
| HC/ YALA -ADR | AGCAAACACGTTGATAGCACAATAACCCTGCTTGGCAATG | to construct vector pGADT7: HC-Pro^O/Y298AL304A^ |
| HC/ YALA-ADF | GCTATCAACGTGTTTGCTGCAATGCTTATTAACATTAGCGAGG |  |
| HC/V346I-ADR | AAGAAAAATGTTGATATAACAATAACCCTGCTTGGCAATG | to construct vector pGADT7: HC-Pro^O/V346I^ |
| HC/V346I-ADF | TATATCAACATTTTTCTTGCAATGCTTATTAACATTAGCGAGG |  |
| HC/N:O-1-ADR | GCTTGCTGCTTCTCCCCTATAGACTTGAATAC | to construct vector pGADT7: HC-Pro^N:O-1^ |
| HC/N:O-1-ADF | GGGAGAAGCAGCAAGCACCGTTCAAGAATT |  |
| HC/N:O-2-ADR | ATTTCATATGCTGAATAGCCCTTCGCTG | to construct vector pGADT7: HC-Pro^N:O-2^ |
| HC/N:O-2-ADF | ATTCAGCATATGAAATCCGCAAGCATCCAAATG |  |
| HC/N:O-3-ADR | TTGATATAACAGAAACCTTGCCTGGCAA | to construct vector pGADT7: HC-Pro^N:O-3^ |
| HC/N:O-3-ADF | GTTTCTGTTATATCAACGTGTTTCTTGCAATGC |  |
| HC/N:O-4-ADR | TTAATGTAACAATAACCCTGCTTGGCAATG | to construct vector pGADT7: HC-Pro^N:O-4^ |
| HC/N:O-4-ADF | GTTATTGTTACATTAACATTTTCCTCGCGATGT |  |

**Table S6. Primers for constructing Co-IP, LUC, and subcellular localization vectors.**

| **Primer name** | **Sequence (5'-3')** | **Note** |
| --- | --- | --- |
| StEXA1-CMycF | ATTACGCCGAGGTCATGGGTGACAAAACTCAATTTGATTCTCG | to construct vector pH7LIC9.0-C-Myc: StEXA1 |
| StEXA1-CMycR | TAGGGAAGAGGGTCTTCCACAGTCTGAATCTCTC |  |
| HC/O-NMycF | ATTACGCCGAGGTCATGTCGAATGCTGATAATTTTTGGAAGG | to construct vector pH7LIC9.0-N-Myc: HC-Pro^O^ |
| HC/O-NMycR | TAGGGAAGAGGTCTCAACCAACTCTATAATGTTTTATATCAGA |  |
| HC/N-NMycF | ATTACGCCGAGGTCATGTCAAGCGCTGAAAGCTTTTGGA | to construct vector pH7LIC9.0-N-Myc: HC-Pro^N^ |
| HC/N-NMycR | TAGGGAAGAGGTCTCAACCAACTCTATAGTGCTTAATGTCAGAC |  |
| StnCBP-CHAF | GGAGATATAACATTACGCCGAGGATGGAAGTGACACCGGAGAAGA | to construct vector pH7LIC7.0-C-HA : StnCBP |
| StnCBP-CHAR | TAACGCGACATATAGGGAAGAGGTGATCTTATCCAAGTATTGCGGTAAG |  |
| GFP-NMycF | ATTACGCCGAGGTCATGGTGAGCAAGGGCGAGGA | to construct vector pH7LIC9.0-N-Myc: GFP |
| GFP-NMycR | TAGGGAAGAGGTCTCACTTGTACAGCTCGTCCATGCC |  |
| StEXA1/AA-CMycR | GCCATTTTTATCCGACTGGCTTGTATAGGAGAGGAAACATTTTCAGCT | to construct vector pH7LIC9.0-C-Myc: StEXA1^Y298AL304A^ |
| StEXA1/AA-CMycF | GCCAGTCGGATAAAAATGGCTGATGTGTATAGAGTTACAGACATGCAG |  |
| nCBP-NLUCF | cgggggacgagctcggtacc ATGGAAGTGACACCGGAGAAGA | to construct vector StnCBP-HA-NLuc |
| nCBP-NLUCR | gcgtacgagatctggtcgac TTATGATCTTATCCAAGTATTGCGGTAAG |  |
| CLUC-OHCF | acgcgtcccggggcggtacc ATGTCGAATGCTGATAATTTTTGGAAGG | to construct vector CLuc-GFP-HC-Pro^O^ |
| CLUC-OHCR | cgaaagctctgcaggtcgac TCAACCAACTCTATAATGTTTTATATCAGA |  |
| CLUC-NHCF | acgcgtcccggggcggtacc ATGTCAAGCGCTGAAAGCTTTTGGA | to construct vector CLuc-GFP-HC-Pro^n^ |
| CLUC-NHCR | cgaaagctctgcaggtcgac TCAACCAACTCTATAGTGCTTAATGTCAGAC |  |
| StEXA1-GFPF | CTCGGCATGGACGAGCTGTACAAGATGGGTGACAAAACTCAATTTGATTCT | to construct vector pK7WGF2: StEXA1 |
| StEXA1-GFPR | CGGGATATCACCACTTTGTACACTAGTCTTCCACAGTCTGAATCTCTC |  |
| StnCBP-GFPF | CTCGGCATGGACGAGCTGTACAAGATGGAAGTGACACCGGAGAAGA | to construct vector pK7WGF2: StnCBP |
| StnCBP-GFPR | CGGGATATCACCACTTTGTACATTATGATCTTATCCAAGTATTGCGGTAAG |  |
| HC/O-GFPF | CTCGGCATGGACGAGCTGTACAAGATGTCGAATGCTGATAATTTTTGGAAG | to construct vector pK7WGF2: HC-Pro^O^ |
| HC/O-GFPR | CGGGATATCACCACTTTGTACATCAACCAACTCTATAATGTTTTATATCAGA |  |
| HC/N-GFPF | CTCGGCATGGACGAGCTGTACAAGATGTCAAGCGCTGAAAGCTTTTGGA | to construct vector pK7WGF2: HC-Pro^N^ |
| HC/N-GFPR | CGGGATATCACCACTTTGTACATCAACCAACTCTATAGTGCTTAATGTCAGA |  |
| StEXA1-RFPF | CGATATCACAAGTTTGTACATGGGTGACAAAACTCAATTTGATTCT | to construct vector pK7WGR2: StEXA1 |
| StEXA1-RFPR | GGATATCACCACTTTGTACCTAGTCTTCCACAGTCTGAATCTCTC |  |
| StnCBP-RFPF | CGATATCACAAGTTTGTACATGGAAGTGACACCGGAGAAGA | to construct vector pK7WGR2: StnCBP |
| StnCBP-RFPR | GGATATCACCACTTTGTACTTATGATCTTATCCAAGTATTGCGGTAAG |  |
| HC/O-RFPF | CGATATCACAAGTTTGTACATGTCGAATGCTGATAATTTTTGGAAGG | to construct vector pK7WGR2: HC-Pro^O^ |
| HC/O-RFPR | GGATATCACCACTTTGTACTCAACCAACTCTATAATGTTTTATATCAGA |  |
| NbDCP1-RFPF | CGATATCACAAGTTTGTACATGTCACAGAACGGAAAATTAATGCC | to construct vector pK7WGR2: NbDCP1 |
| NbDCP1-RFPR | GGATATCACCACTTTGTACTTATGAATGATGAGCATTTAGCACTGCC |  |
| NbUBP1-RFPF | CGATATCACAAGTTTGTACATGATGCAGCAGAGGCTGAAG | to construct vector pK7WGR2: NbUBP1 |
| NbUBP1-RFPR | GGATATCACCACTTTGTACCTAGTACATCGATGGTTGAGTTGTAGC |  |


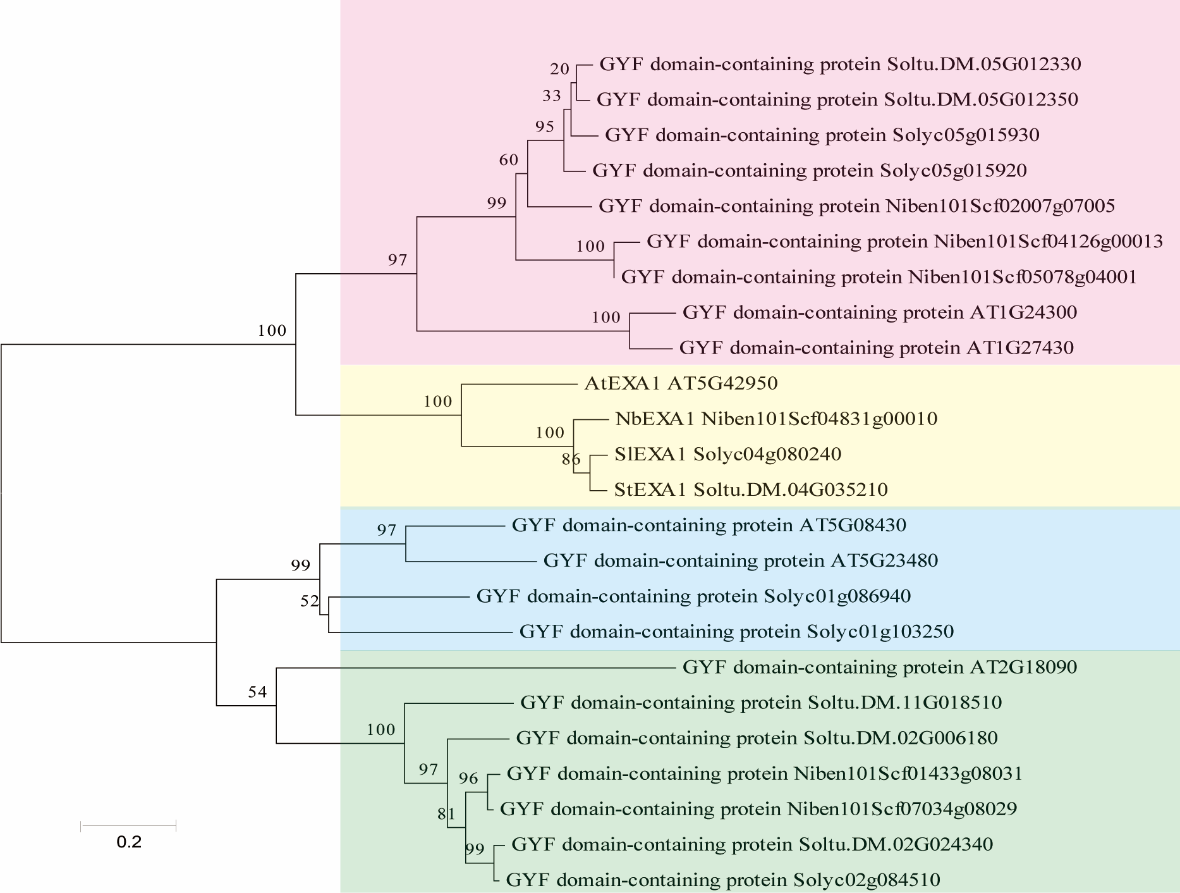


**Fig. S1 Phylogenetic analysis of amino acid sequences of EXA1 and other GYF domain-containing proteins from *S.tuberosum, A.thaliana, N.benthamiana* and *S.lycopersicum****.* The prefix of accession numbers with ‘Soltu.DM’ represent proteins from *S. tuberosum*; the prefix of accession numbers with ‘AT’ represent proteins from *A. thaliana*; the prefix of accession numbers with ‘Solyc’ represent proteins from *S. lycopersicum*; the prefix of accession numbers with ‘Niben101Scf’ represent proteins from *N. benthamiana.* The proteins are divided into four groups marked with different colors. The phylogenetic tree was generated with Neighbor-Joining Tree and 1000 bootstraps by MEGA5.2.


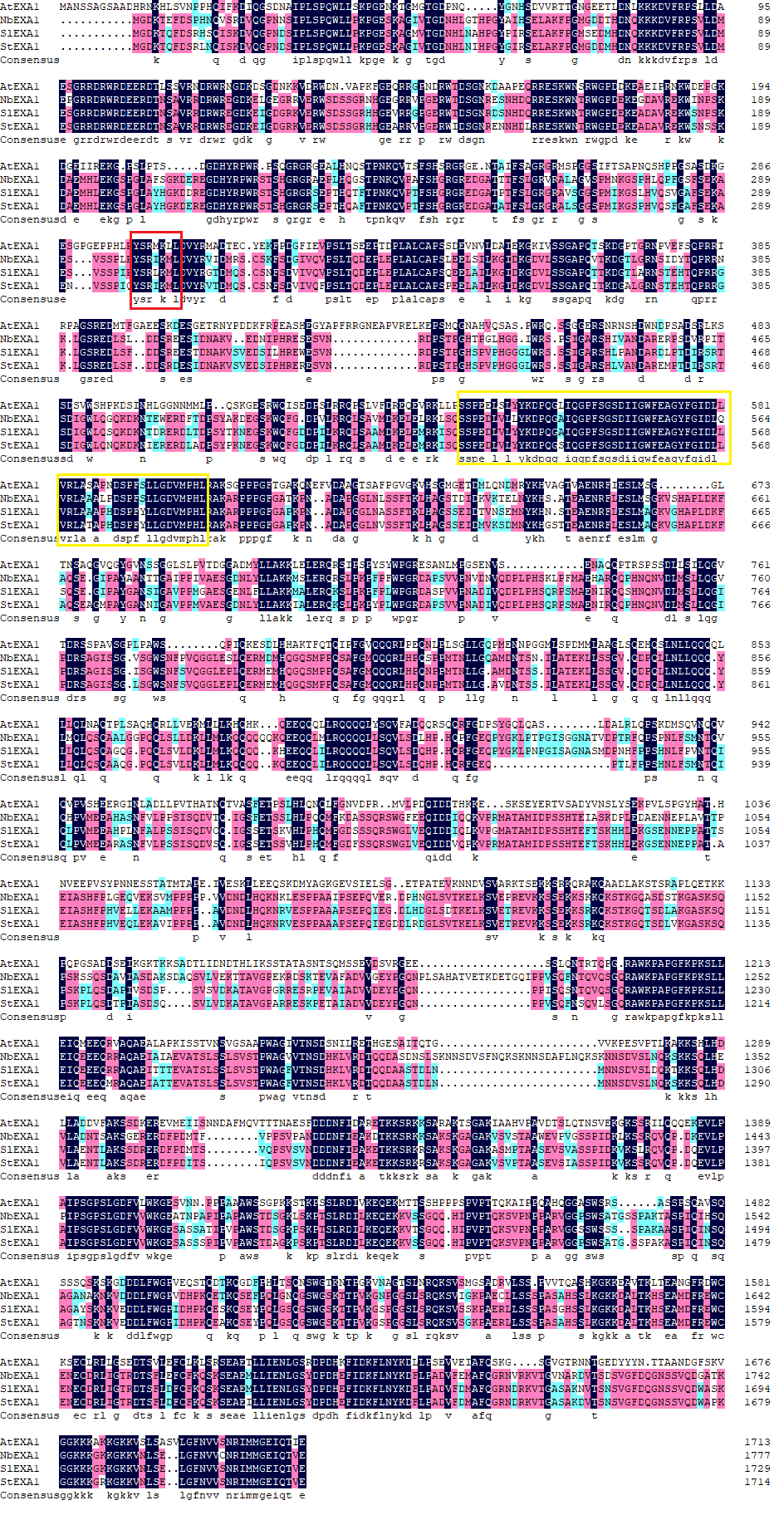


**Fig. S2 Amino acid sequences are aligned for EXA1 proteins from four species.** StEXA1, AtEXA1, SlEXA1, and NbEXA1 represent EXA1 proteins from *S. tuberosum, A.thaliana*, *S.lycopersicum*, and *N.benthamiana* respectively. The eIF4E-binding motif was marked with a red box and the GYF domain was marked with a yellow box.


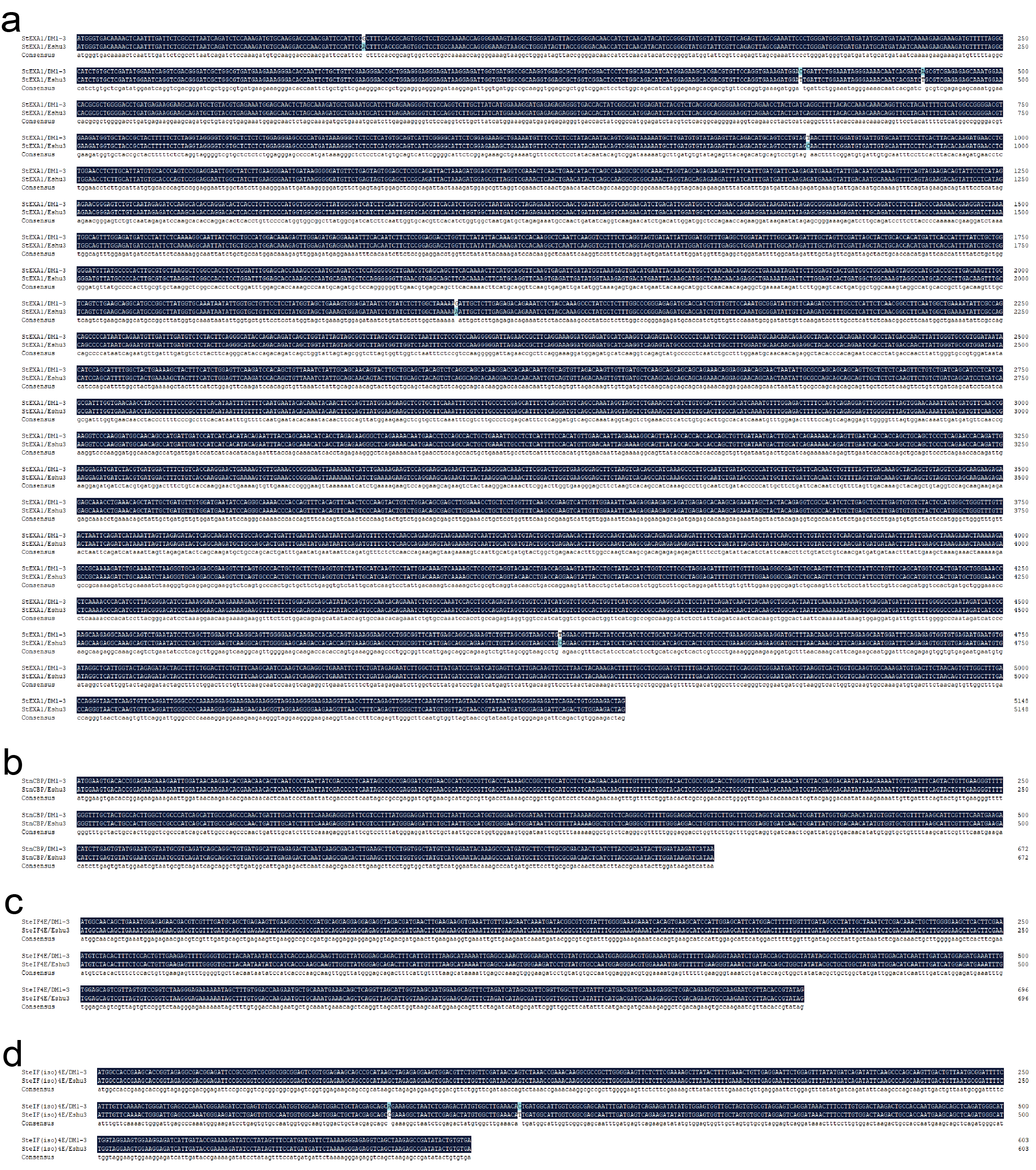


**Fig. S3 Nucleotide sequences alignment of *StEXA1* (a)**, ***StnCBP* (b)*,* *SteIF4E* (c), and *SteIF(iso)4E* (d)** **in DM1-3 and Eshu3.** The nucleotide sequences of S*tEXA1*, *StnCBP, SteIF4E* and *SteIF(iso)4E* in DM1-3 show 99.88%, 100%, 100%, 99.67% identitie with those in Eshu3, respectively.


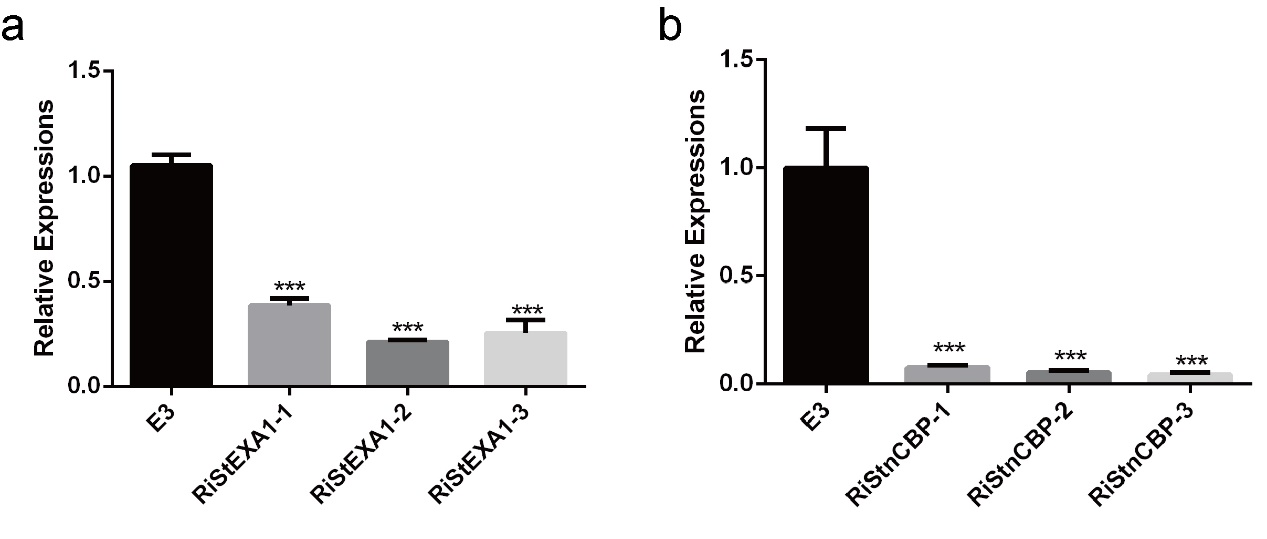


**Fig. S4 Interference efficiency of RiStEXA1 (a) and RiStnCBP (b) transgenic lines.** The expression of each gene were determined by qRT-PCR. Data are presented as means ± SD (n=3) relative to WT (E3) plants, and *EF1α* was used as the normalizer. Asterisks indicate statistically significant differences according to Student’s t-test (p < 0.001 ***).

**
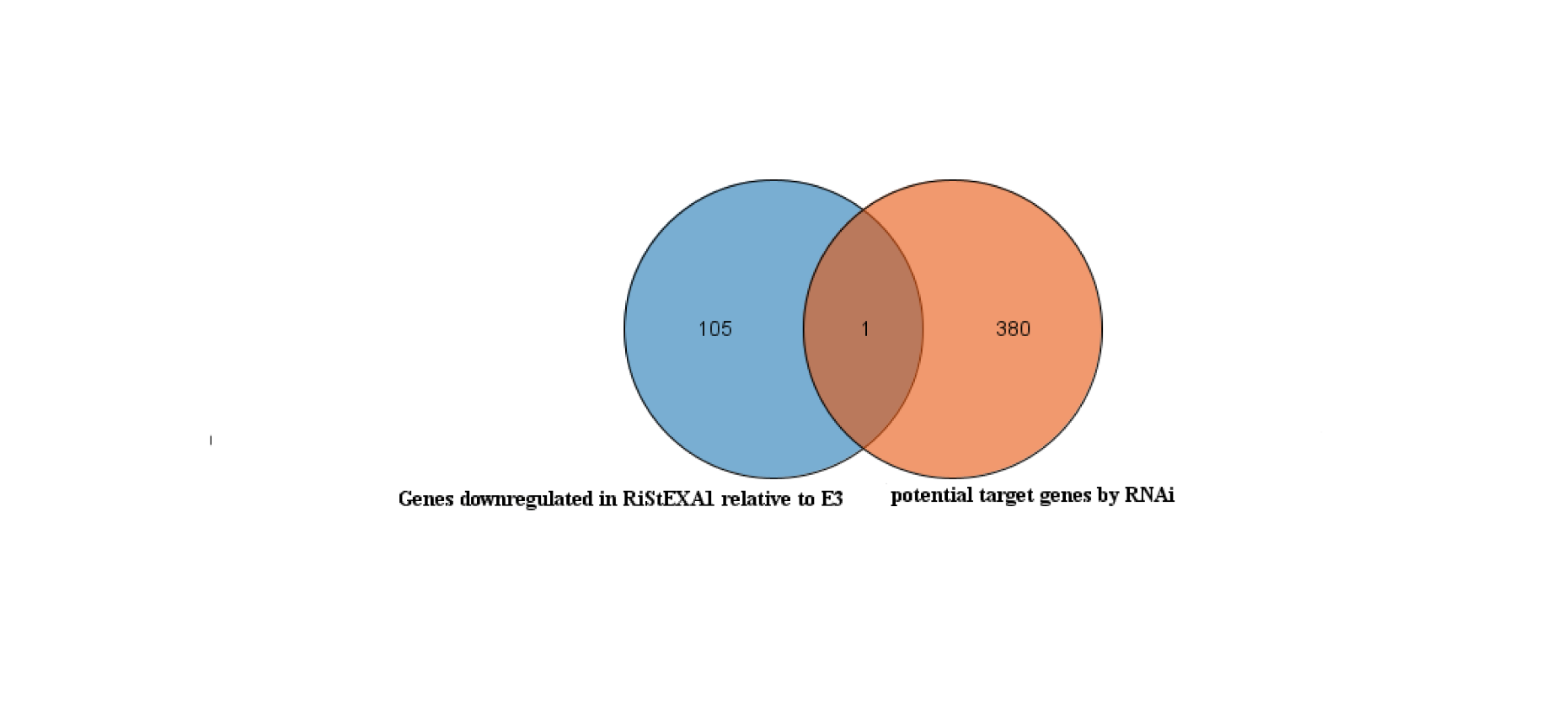
**

**Fig. S5 Venn diagram showing the intersection of potential targets of RNAi and the down-regulated genes in RiStEXA1 lines.** The intersection contains one gene StEXA1 ([Soltu.DM.04G035210](http://spuddb.uga.edu/cgi-bin/annotation_report.cgi?orf=Soltu.DM.04G035210)).


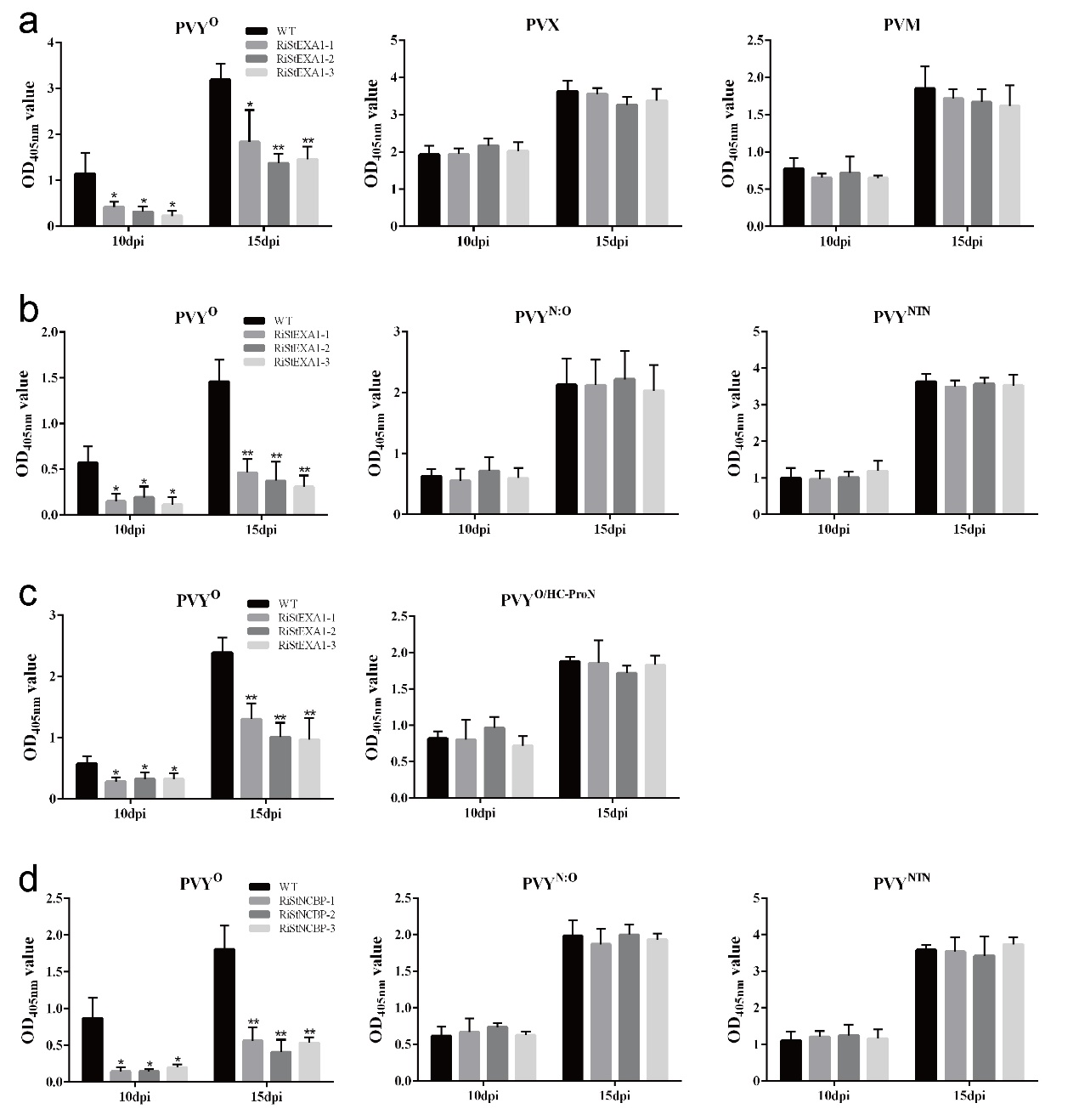


**Fig. S6 Enzyme-linked immunosorbent assay (ELISA) detects the viruses accumulation in the leaves of transgenic and control plants (WT).** (**a**) ELISA result of potato *RiStEXA1* and control plants inoculated with PVY^O^, PVX, and PVM. (**b**) ELISA result of *RiStEXA1* and control plants inoculated with three PVY strains. (**c**) ELISA result of *RiStEXA1* and control plants inoculated with PVY^O^ and PVY^O^ with HC-Pro^N^. (**d**) ELISA result of *RiStnCBP* and control plants inoculated with three PVY strains. Plants were mechanically inoculated with viruses and non-inoculated upper leaves were collected at 10 and 15 dpi for ELISA. The ELISA values are presented as means ± SD (n=3). Three independent experiments were performed with similar results. Asterisks indicate statistically significant differences according to Student’s t-test (p < 0.01 **, p < 0.05 *).


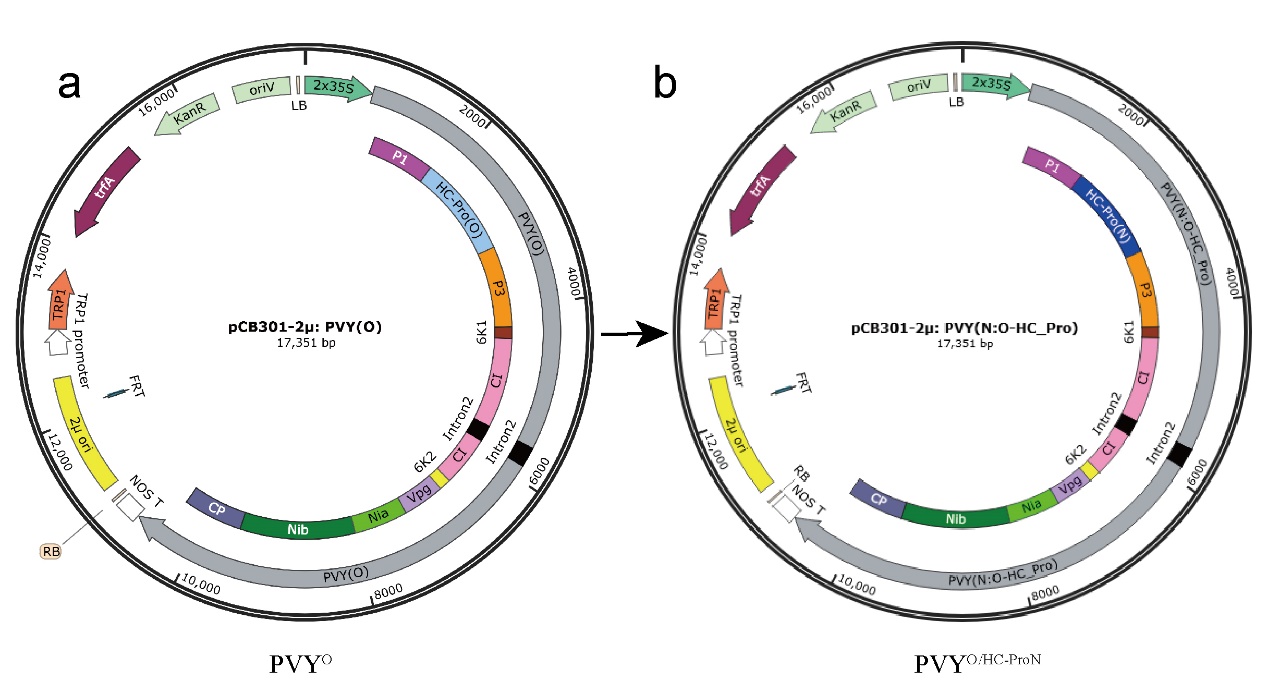


**Fig. S7 Schematic illustration shows the constructions of infectious cDNA clones for PVY^O^ (a) and** **PVY^O/HC-ProN^ (b).** pCB301-2μ-PVY^O^ was constructed as the method described previously81. The gray bar indicates the insertion position of PVY while Intron2 indicates the insertion position of *NIR* intron2 (Accession number: U10419.1).


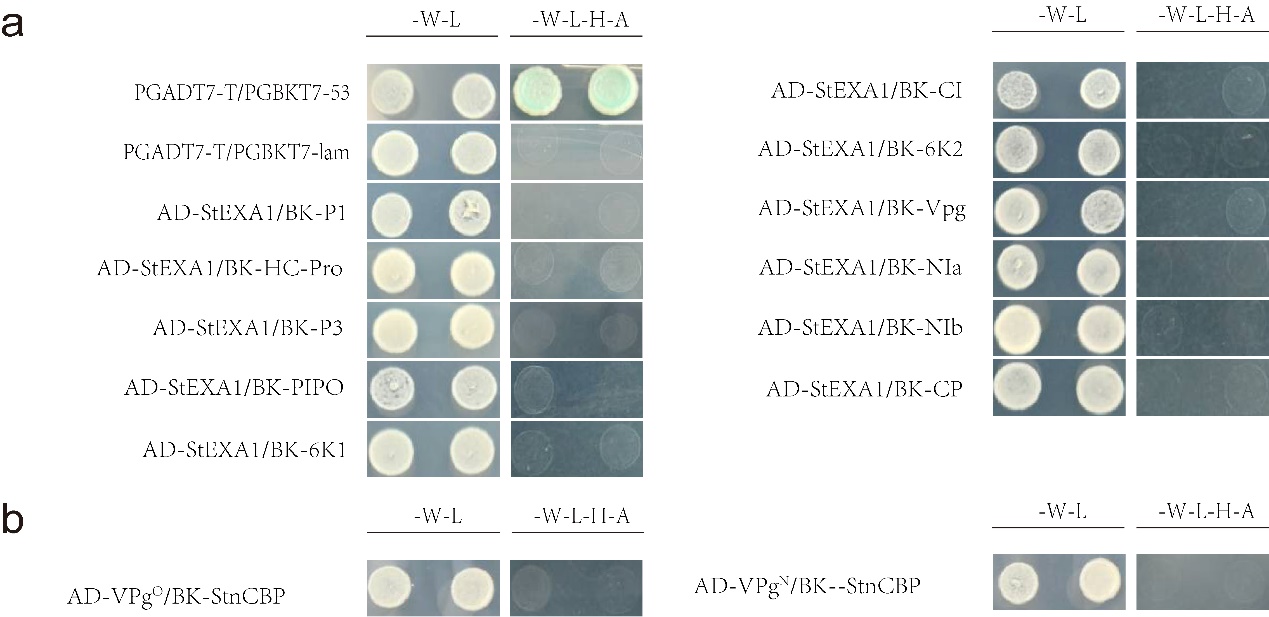


**Fig. S8 StEXA1 exhibits no interaction with any of the 11 proteins of PVY^O^ in Y2H assays (a) and StnCBP exhibits no interaction with VPg of PVY (b).** -W-L represents medium lacking tryptophan and leucine, -W-L-H-A represents medium lacking tryptophan, leucine, histidine, and adenine. Paired combinations PGADT7-T/PGBKT7-53 and PGADT7-T/PGBKT7-lam represent positive and negative controls, respectively.


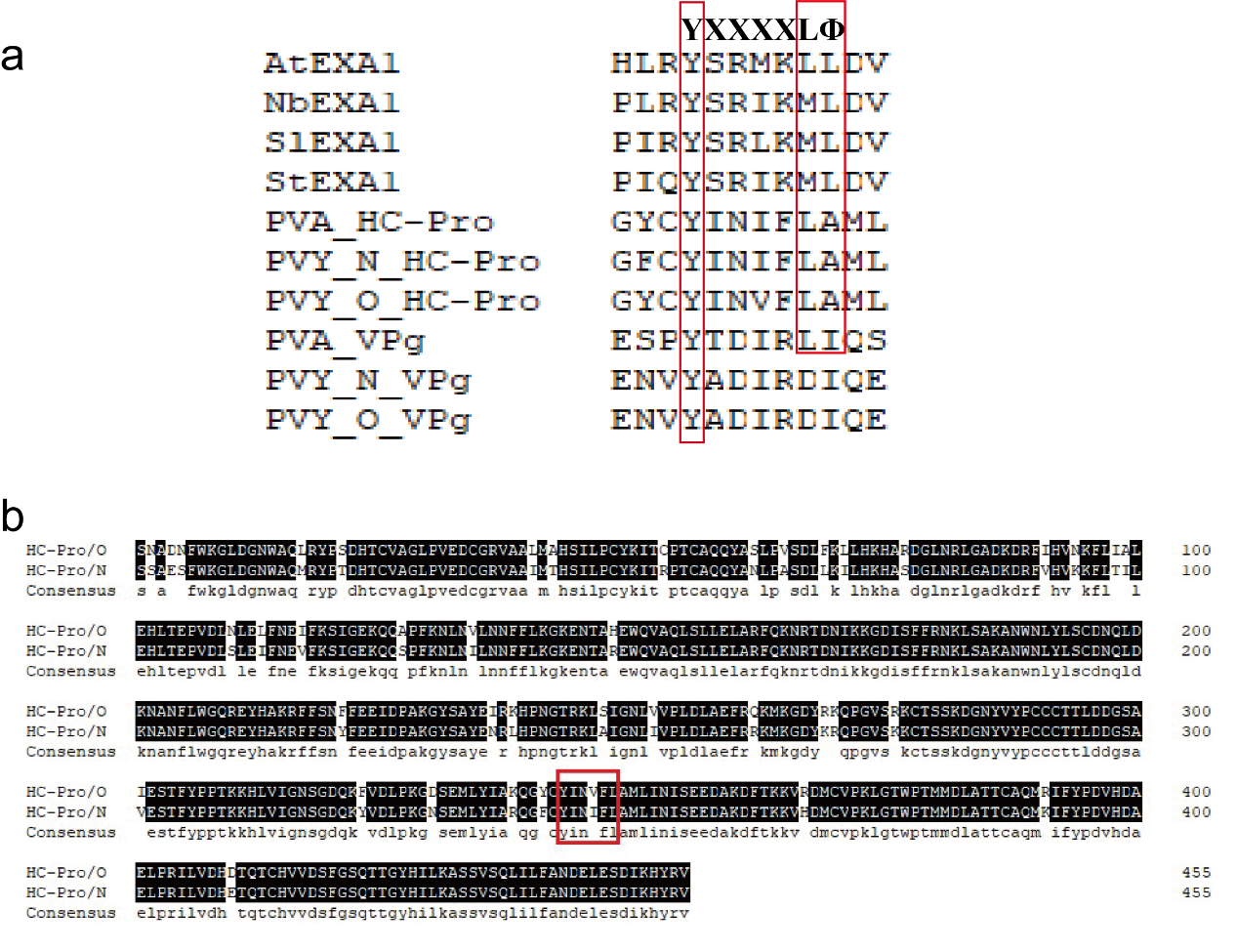


**Fig. S9 Alignment of eIF4E-binding motif on EXA1, HC-Pro and VPg proteins (a), and amino acid sequences alignment of HC-Pro proteins from PVY^O^ and PVY^N^ (b).** In **(a)**, AtEXA1, SlEXA1, and NbEXA1 and StEXA1 represent eIF4E-binding motifs on EXA1 proteins of *A. thaliana*, *S. Lycopersicum*, and *N. benthamiana* respectively. PVA_HC-Pro and PVA_VPg represent eIF4E-binding motifs on HC-Pro and VPg proteins of PVA respectively. PVY_HC-Pro/O and PVY_HC-Pro/N represent eIF4E-binding motifs on HC-Pro of PVY^O^ and PVY^N^ respectively. The conserved amino acid residues on eIF4E-binding motifs were marked with red boxs. In **(b)**, HC-Pro/O and HC-Pro/N represent HC-Pro proteins from PVY^O^ and PVY^N^, respectively. The eIF4E-binding motif was marked with a red box.
